# HCMV US30 induces TMED10-DRP1-mediated mitochondrial fission and apoptosis to promote viral multiplication

**DOI:** 10.1128/mbio.01740-26

**Published:** 2026-09-22

**Authors:** Ya-Li Lei, Yu-Ting Cai, Ruo-Tong Chen, Peng Ren, Xue-Mei Yi, Yu-Zhi Fu, Yan-Yi Wang, Shu Li, Hong-Bing Shu

**Affiliations:** 1 Department of Infectious Diseases, Zhong nan Hospital of Wuhan University; State Key Laboratory of Virology and Biosafety, Hubei Provincial Research Center for Basic Biological Sciences, Frontier Science Center for Immunology and Metabolism, Medical Research Institute, Taikang Center for Life and Medical Sciences, Wuhan University 555074, Wuhan, China; 2 State Key Laboratory of Virology and Biosafety, Wuhan Institute of Virology, Chinese Academy of Sciences 74614, Wuhan, Hubei, China; Tsinghua University, Beijing, China

**Keywords:** HCMV, US30, apoptosis, UPR, mitochondrial fission, viral replication

## Abstract

Human cytomegalovirus (HCMV) hijacks host components to modulate cell death for its optimized replication and dissemination. We identified HCMV US30 as a viral factor that induces caspase-dependent apoptosis. US30 induces both DRP1-dependent mitochondrial fission and subsequent apoptosis, as well as IRE1α-mediated unfolded protein responses (UPR). Mechanistically, US30 interacts with NPL4 to impair ER-associated protein degradation and subsequently induce UPR. In parallel, US30 associates with the Endoplasmic reticulum-Golgi intermediate compartment (ERGIC) protein TMED10 and promotes its translocation to the cytoplasm, where it activates DRP1 to initiate mitochondrial fission and subsequent caspase-dependent apoptosis. US30 induces UPR and mitochondrial fission/apoptosis via distinct domains, and the two distinct effects are uncoupled. We also show that US30-induced mitochondrial fission and apoptosis, but not UPR, contribute to the multiplication and dissemination of HCMV. These findings reveal that HCMV US30 induces mitochondrial fission and apoptosis to promote viral replication and dissemination and provide potential targets for intervention of HCMV-induced diseases.

**IMPORTANCE:**

Human cytomegalovirus (HCMV) is a human pathogenic herpesvirus that causes global epidemics. HCMV hijacks cellular compartments to maintain its replication and dissemination, which is associated with host cell fate. Here, we identified HCMV US30 as a viral factor to induce caspase-dependent apoptosis. Proteomics analysis revealed that US30 disrupts both ER and mitochondrial homeostasis. We further found that US30-induced mitochondrial fission, but not UPR, mediates apoptosis. Mechanistically, our results indicate that US30 interacts with NPL4 to activate UPR and associates with TMED10 to trigger mitochondrial fission, which are dependent on distinct domains of US30. Most importantly, we found that US30 alters the localization of TMED10 from ERGIC to the cytosol to activate DRP1. Finally, our results showed that US30-induced mitochondrial fission and apoptosis, but not UPR, contribute to the multiplication and dissemination of HCMV. These findings enhance our understanding of HCMV-host interplay and provide potential targets for intervention of HCMV-induced diseases.

## INTRODUCTION

Human cytomegalovirus (HCMV) is a highly prevalent β-herpesvirus that establishes lifelong persistence in the human population (1). While primary infection is typically subclinical and asymptomatic in immunocompetent individuals, HCMV can cause severe diseases in immunocompromised patients and newborns, such as birth defects, pneumonia, hepatitis, retinitis, and is associated with various cancers and chronic illnesses (2). Productive HCMV infection requires extensive reprogramming and hijacking of host cellular processes to create a favorable environment for viral replication, dissemination, and persistence, which are tightly coupled to host cell fate decisions (3–5). In this context, modulation of host cell death emerges as a key determinant for sustained viral replication and dissemination. Suppression of premature death of host cells enables efficient viral genome replication, whereas induction of cell death at later stages of infection facilitates the release and dissemination of progeny virions (6–8).

Consistent with critical roles of cell death in regulation of viral life cycles, HCMV has evolved diverse strategies to promote its multiplication via manipulating host cell death (9–11). UL37x1 (vMIA, viral mitochondrial inhibitor of apoptosis) prevents mitochondrial outer membrane permeabilization from initiating intrinsic apoptosis to ensure efficient virus replication (7, 9). UL36 (vICA, viral inhibitor of caspase-8-induced apoptosis) inhibits caspase-8 activation to abrogate extrinsic apoptotic signaling, supporting effective HCMV replications (10, 12). In addition, viral microRNAs such as miR-US5-1 and miR-UL112-3p suppress the expression of the proapoptotic BCL2L11 in CD34+ HPCs, preventing their apoptosis and allowing for the establishment of latency and maintenance of infected cells (11). There are also reports that HCMV encodes proteins to induce cell death, such as US28, UL13, and US18, which induce apoptosis, cuproptosis, and pyroptosis, respectively (13–15). Although the effects of cell death on HCMV replication and dissemination remain less known, there are reports that cell death is utilized by other herpesviruses to promote virus egress or replication (8, 16).

It has been demonstrated that cell death induced by infection of certain viruses can be mediated by cellular stress responses, such as induction of ER-associated protein degradation (ERAD) and unfolded protein response (UPR), two conserved branches of ER quality-control mechanisms (17). ERAD targets unfolded or misfolded proteins for ubiquitin-dependent degradation, while UPR is activated in response to impaired ERAD via three sensors including IRE1α, PERK, and ATF6, leading to the activation of downstream transcription factors containing XBP1S, ATF4, and ATF6-C (18, 19). Previously, it has been demonstrated that the p97/VCP-UFD1-NPL4 AAA+ ATPase complex is required for ERAD via extracting unfolded or misfolded proteins for proteasomal degradation (20). NPL4 is considered an adaptor of p97 by forming a heterodimer with UFD1, recognizing ubiquitinated ERAD substrates and directly delivering them to p97 for further degradation by the 26S proteasomes (21). Persistent disruption of ERAD process leads to the accumulation of unfolded or misfolded proteins, triggering caspase-12-dependent cell death (22). In parallel, mitochondrial dynamics has emerged as one of the key determinants of intrinsic apoptosis (23), among which mitochondrial fission, primarily mediated by the dynamin-related GTPase DRP1, has been reported to promote mitochondrial outer-membrane permeabilization, mitochondrial fragmentation, and subsequent activation of the intrinsic apoptosis (24). It has been well established that phosphorylation at S616 and dephosphorylation at S637 of DRP1 in the cytosol promotes its translocation to the mitochondrial outer-membrane to initiate mitochondrial fission (25, 26). Previous studies have indicated that both of UL37x1 and UL13 promote mitochondrial fission during early phase of HCMV infection (27–29), and an ER resident glycoprotein UL148 encoded by HCMV activates UPR and stabilize viral envelope glycoprotein complex (30). However, whether additional HCMV-encoded proteins contribute to these processes remains largely unknown.

In this study, we identified HCMV US30 as a viral factor that induces cell death in a systemic screen of HCMV proteins. We show that US30 induces both caspase-dependent apoptosis and IRE1α-mediated UPR. US30 interacts with the p97 ATPase cofactor NPL4 to impair ERAD, resulting in UPR. Additionally, US30 associates with the ERGIC protein TMED10, which promotes the translocation of TMEM10 to cytosol, at where it induces DRP1 S616 phosphorylation and S637 dephosphorylation, leading to mitochondrial fission and subsequent caspase-dependent apoptosis. US30 interacts with NPL4 and TMED10 via distinct domains, and US30-induced UPR and apoptosis are uncoupled. Lastly, we show that US30-induced mitochondrial fission and apoptosis, but not UPR, contribute to the multiplication and dissemination of HCMV. These findings suggest that HCMV US30 modulates mitochondrial fission and apoptosis to promote its multiplication and dissemination, pointing to a potential strategy for intervention of HCMV infectious diseases.

## RESULTS

### US30 mediates HCMV-induced apoptosis

**Fig 1:**
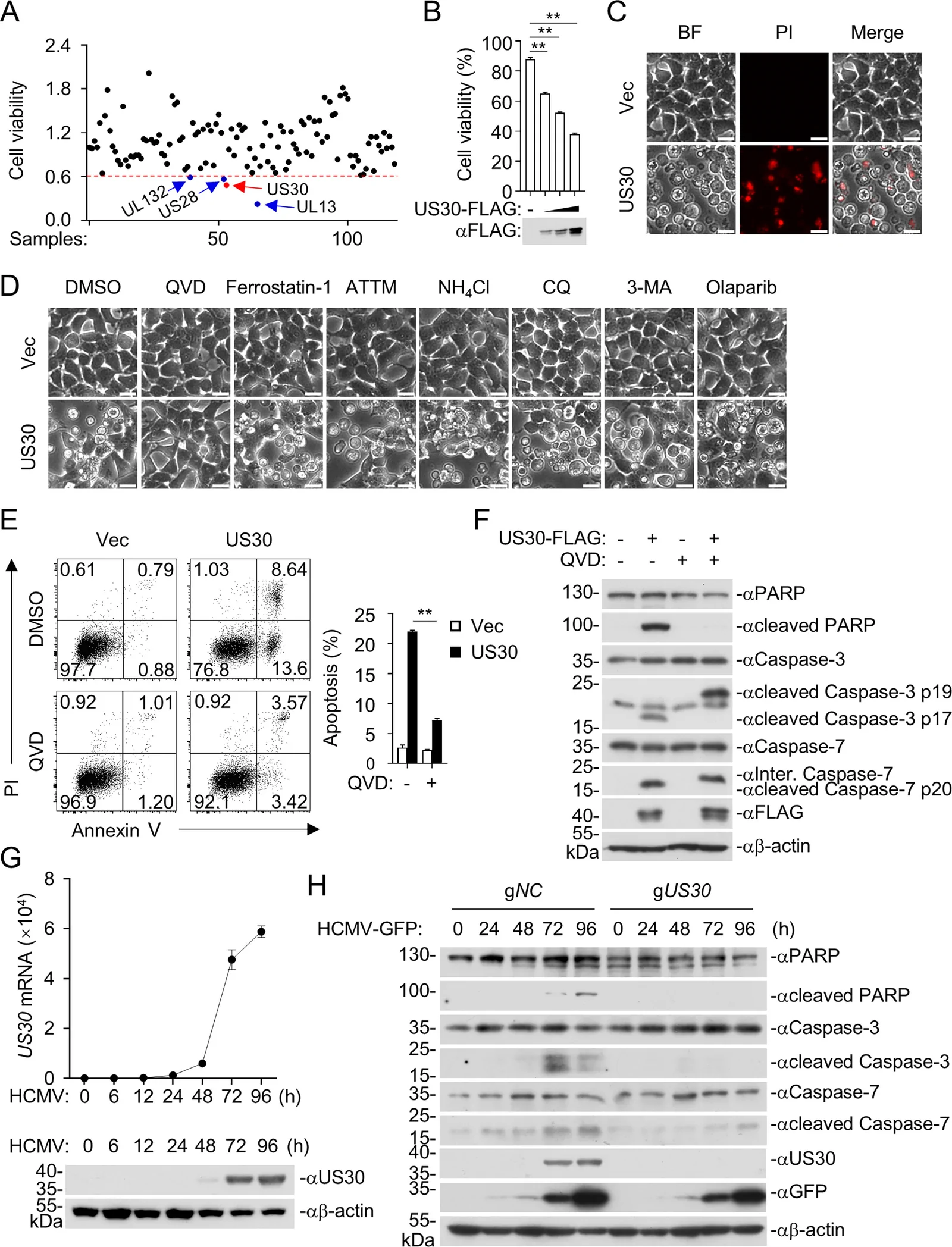
US30 mediates HCMV-induced apoptosis. (**A**) Screening of HCMV-encoded proteins that regulate cell viability. HEK293 cells (5 × 10^3^) were seeded into 96-well plates and transfected with HCMV-encoded proteins (50 ng) or empty vector for 36 h before CellTiter-Glo assays. Data were normalized to empty vector-transfected control. Among the candidates, US30 was labeled red, and the other three candidates were labeled blue. (**B**) US30 inhibits cell viability in a dose-dependent manner. HEK293 cells (5 × 10^3^) were seeded into 96-well plates and transfected with US30-FLAG plasmid (0, 30, 60, 90 ng) or empty vector for 24 h before CellTiter-Glo assays. The empty vector was supplemented to maintain a constant total DNA amount in each transfection. (**C**) US30 induces cell death. HEK293 cells (5 × 10^5^) were transfected with US30-FLAG plasmid (0.5 μg) or empty vector for 24 h before propidium iodide (PI) staining and fluorescence microscopy assays. Scale bars, 20 μm. (**D**) Effects of distinct inhibitors on US30-induced cell death. HEK293 cells (5 × 10^5^) were transfected with US30-FLAG plasmid (0.5 μg) or empty vector for 4 h followed by treatment with the indicated inhibitors: QVD (20 μM), ferrostatin-1 (20 μM), ATTM (40 μM), NH_4_Cl (10 mM), CQ (40 μM), 3-MA (0.5 mM), and olaparib (10 μM). The cells were subsequently visualized by light microscopy at approximately 24 h post-transfection. Scale bars, 20 μm. (**E**) Measurement of US30-induced cell death by annexin V-FITC/PI staining. HEK293 cells (5 × 10^5^) were transfected with US30-FLAG plasmid (0.5 μg) or empty vector for 4 h followed by treatment with DMSO or QVD (20 μM) for 20 h. The cells were collected and stained by annexin V-FITC/PI-PerCP-Cy5.5 for flow cytometry analysis (left) (Q2: annexin V and PI double positive, late apoptosis; Q3: annexin V single positive, early apoptosis), and the percentage of apoptotic cells (Q2 + Q3) was measured (right). (**F**) Effects of US30 on the cleavage of PARP and caspase-3/7. HEK293 cells (5 × 10^5^) were transfected with US30-FLAG plasmid (0.5 μg) or empty vector for 4 h followed by treatment with DMSO or QVD (20 μM) for 20 h before immunoblotting analysis with the indicated antibodies. (**G**) Expression of US30 following HCMV infection. HFFs (5 × 10^5^) were left uninfected or infected with HCMV AD169 (MOI = 1) for the indicated times before qPCR assays and immunoblotting analysis with the indicated antibodies. (**H**) Effects of US30-deficiency on HCMV-induced cleavage of PARP and caspase-3/7. The control and *US30* gRNA-edited HFFs (5 × 10^5^) were left uninfected or infected with HCMV-GFP (MOI  =  1) for the indicated times before immunoblotting analysis with the indicated antibodies. The data shown in panels **B**, **E,** and **G** are represented as mean ± SEM from one representative experiment, *n*  =  4 independent samples in panel **B**, *n* = 3 independent samples in panels **E** and **G**. Panels **B–H** were repeated for at least two times with similar results. n.s., not significant. *P  <  0.05, **P  <  0.01.

To identify HCMV proteins that induce cell death, 118 expression clones encoding HCMV (AD169 strain) proteins were screened by CellTiter-Glo assays. These screens identified four clones, which encode UL13, US28, US30, and UL132, respectively, and decrease cell viability to below 60% relative to the empty control vector upon transfection in HEK293 cells (Fig. 1A). Among the four candidate clones, US28 and UL13 have been previously reported to induce extrinsic apoptosis or cuproptosis, respectively (13, 14). In this study, we have focused on investigation of a potential role of US30 in cell death. We first validated that ectopic expression of US30 reduced cell survival in a dose-dependent manner in CellTiter-Glo assays (Fig. 1B). Ectopic expression of US30 induced dramatic morphological changes of typical cell death and uptake of propidium iodide (PI) into the cells, suggesting that US30 induces cell death (Fig. 1C). Notably, although the majority of cells appeared predominantly rounded, shrunken, and less adherent after transfection with US30, only a fraction of these “dead” cells were labeled by PI, suggesting the population of “dead” cells comprises cells with either intact or disrupted plasma membrane.

To determine the types of cell death induced by US30, we treated the cells with various cell death inhibitors after transfection of US30 for 4 h, including Q-VD-Oph (QVD, a pan-caspase apoptosis inhibitor), ferrostatin-1 (a ferroptosis inhibitor), the copper chelator ammonium tetrathiomolybdate (ATTM, a cuproptosis inhibitor), olaparib (a PARP inhibitor), NH_4_Cl, chloroquine (CQ), and 3-MA (autophagy inhibitors). The results indicated that only QVD blocked US30-induced cell death, suggesting that US30 induces caspase-dependent cell death (Fig. 1D). Flow cytometry analysis indicated that the overexpression of US30 obviously increased the ratios of annexin V- or annexin V- and PI-positive populations, and QVD significantly inhibited them (Fig. 1E). Moreover, US30 promoted the production of cleaved PARP, cleaved caspase-3 p17, and cleaved caspase-7 p20, which are hallmarks of apoptosis, and treatment with QVD reduced the production of the cleaved PARP and inhibited the cleavage of caspase-3 p19 into p17 and caspase-7 intermediate products into p20 (Fig. 1F). These data collectively suggest that US30 induces caspase-dependent apoptosis.

We next investigated whether US30 plays a role in apoptosis induced by HCMV. Following HCMV infection, both US30 mRNA and protein were detected at 48 hours post-infection (hpi) and remarkably enriched at 72 hpi (Fig. 1G). We then examined the role of US30 in HCMV-induced apoptosis by CRISPR-Cas9-mediated editing of the *US30* gene. The results indicated that US30-deficiency largely inhibited the cleavage of PARP and caspase-3/7 induced by HCMV infection (Fig. 1H). These results suggest that US30 plays an important role in HCMV-induced apoptosis.

### US30-induced UPR and apoptosis are uncoupled

To find out how US30 induces caspase-dependent apoptosis, we performed proteomics analysis after ectopic expression of US30 in HEK293 cells. Analysis of differentially expressed proteins indicated that ectopic expression of US30 caused strong upregulation of certain ER proteins, as well as dramatic downregulation of mitochondrial matrix components (Fig. S1A). Further analysis of these regulated proteins indicated that US30 induced upregulation of ER proteins involved in UPR and downregulation of mitochondrial proteins involved in oxidative phosphorylation (Fig. S1B). It has been reported that persistent activation of UPR induces caspase-dependent apoptosis, and disruption of mitochondrial functions causes intrinsic apoptosis (31–33). Therefore, we investigated whether US30 induces apoptosis via activation of UPR and/or disruption of mitochondrial functions.

**Fig 2:**
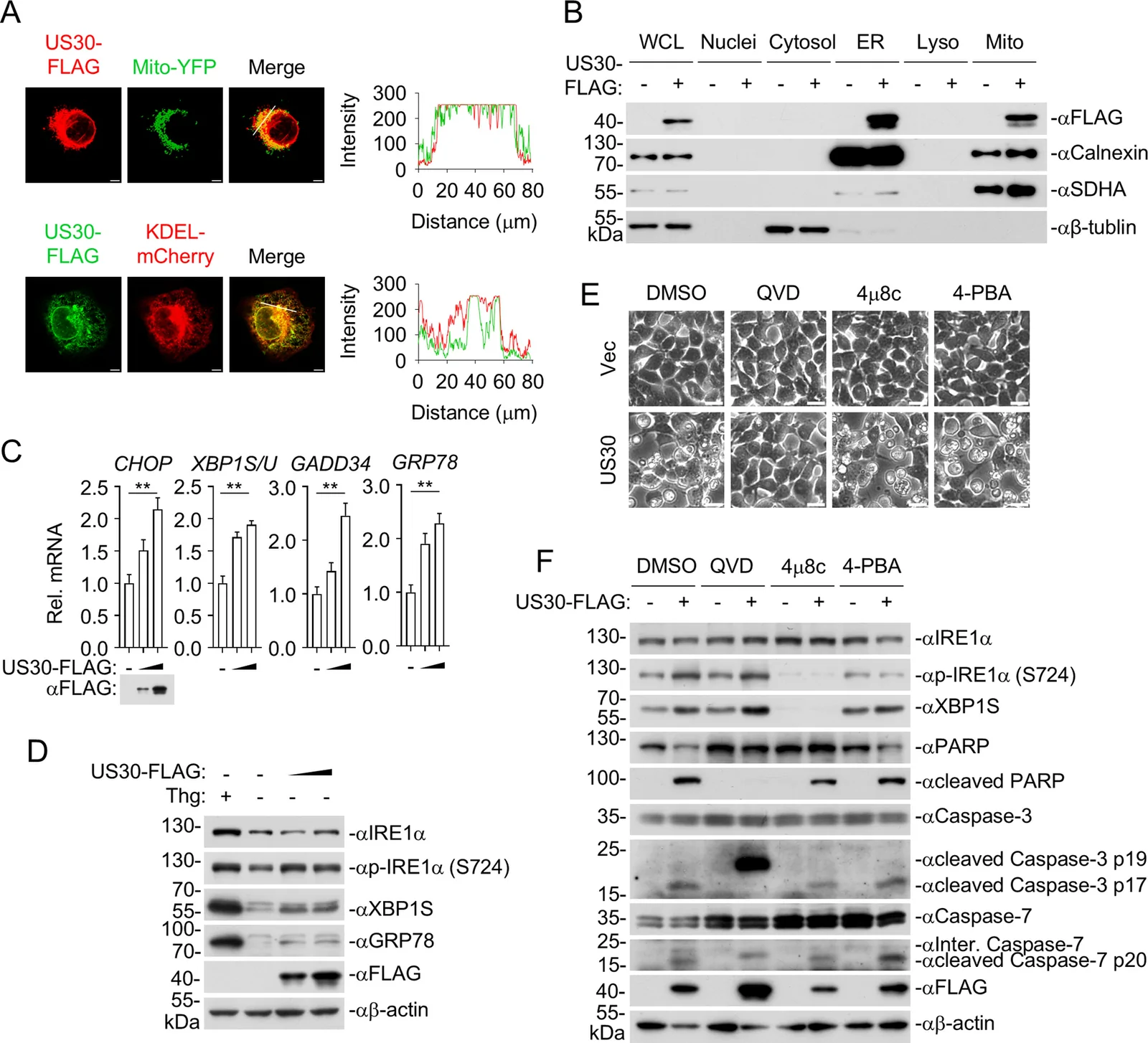
US30-induced UPR and apoptosis are uncoupled (**A**) US30 is localized to ER and mitochondria. HFFs (5 × 10^4^) were transfected with expressing plasmids for US30-FLAG (0.2 μg) and KDEL-mCherry (0.2 μg) or Mito-YFP (0.2 μg) for 24 h before confocal microscopy. Scale bars, 20 μm. Fluorescence intensity profiles were generated by ImageJ. (**B**) Subcellular fractionation analysis of US30. HEK293 cells (2 × 10^7^) were transfected with US30-FLAG plasmid (5 μg) or empty vector for 24 h before subcellular fractionation and immunoblotting analysis with the indicated antibodies. (**C**) Effects of US30 on transcription of downstream genes of UPR. HEK293 cells (5 × 10^5^) were transfected with US30-FLAG plasmid (0, 0.5, 1 μg) or empty vector for 24 h before qPCR analysis. Data shown are mean ± SEM from one representative experiment, *n* = 3 independent samples. n.s., not significant. *P  <  0.05, **P  <  0.01. (**D**) Effects of US30 on levels of UPR-related proteins. HEK293 cells (5 × 10^5^) were transfected with US30-FLAG plasmid (0, 0.5, 1 μg) for 18 h and then left untreated or treated with thapsigargin (2 μM) for 6 h before immunoblotting analysis with the indicated antibodies. (**E**) Effects of IRE1α inhibitor (4μ8c) and ER stress inhibitor (4-PBA) on US30-induced cell death. HEK293 cells (5 × 10^5^) were transfected with US30-FLAG plasmid (0.5 μg) or empty vector for 4 h and then left untreated or treated with QVD (20 μM), 4μ8c (20 μM), or 4-PBA (2 mM). The cells were subsequently visualized by light microscopy at ~ 24 h post-transfection. Scale bars, 20 μm. (**F**) Effects of 4μ8c and 4-PBA on US30-induced UPR and the cleavage of PARP and caspase-3/7. HEK293 cells (5 × 10^5^) were transfected with US30-FLAG plasmid (0.5 μg) or empty vector for 4 h and then left untreated or treated with QVD (20 μM), 4μ8c (10 μM), or 4-PBA (2 mM) for 20 h before immunoblotting analysis with the indicated antibodies. All the experiments were repeated for at least two times with similar results.

First, we examined the subcellular location of US30 in human foreskin fibroblasts (HFFs) by confocal microscopy and subcellular fractionation assays. The results indicated that US30 was located to both the ER and mitochondria (Fig. 2A and B). We next evaluated the effects of US30 on ER homeostasis. The results indicated that ectopic expression of US30 enhanced ER signal and cytosolic Ca^2+^ concentration (Fig. S1C and D), which are hallmarks of ER stress and UPR (34). Upon ER stress, the three sensors, including IRE1α, PERK, and ATF6, dissociate with GRP78 (35). The dissociation of IRE1α with GRP78 leads to the phosphorylation of IRE1α at S724, resulting in unconventional splicing of XBP1 into XBP1S and subsequent ERAD. The disassociation of PERK with GRP78 allows its autophosphorylation and induction of ATF4-dependent transcription of the stress genes including *CHOP* and *GADD34*, leading to the inhibition of protein translation. The dissociation of the transcription factor ATF6 with GRP78 leads to its cleavage in the Golgi to generate an active transcription factor that drives the expression of genes involved in ER quality control and remodeling (35). The three branches of UPR are highly interconnected with shared downstream targets (36, 37). Quantitative PCR (qPCR) experiments indicated that ectopic expression of US30 upregulated the downstream genes of UPR, including *CHOP*, *XBP1*, *GADD34*, and *GRP78* (Fig. 2C). In addition, similar to the UPR inducer thapsigargin, ectopic expression of US30 enhanced the levels of p-IRE1α, GRP78, and XBP1S, which are hallmarks of UPR activation (Fig. 2D). These results suggest that US30 induces UPR.

We next investigated whether US30 induces caspase-dependent apoptosis via activating UPR. We examined whether treatment with an IRE1α endoribonuclease inhibitor (4μ8c) or an ER stress inhibitor (4-phenylbutyric acid [4-PBA]) inhibits cell death induced by US30. The results indicated that both 4μ8c and 4-PBA inhibited US30-induced increase of the levels of p-IRE1α(S724) and XBP1S but failed to inhibit US30-induced cell death (Fig. 2E) and cleavage of PARP and caspase-3/7 (Fig. 2F). In these experiments, the caspase inhibitor QVD inhibited US30-induced cell death, and cleavage of PARP and caspase-3/7, but did not inhibit the levels of p-IRE1α(S724) and XBP1S (Fig. 2E and F). These results suggest that US30-induced IRE1α-mediated UPR and apoptosis are uncoupled.

### US30 induces mitochondrial fission to initiate apoptosis

**Fig 3:**
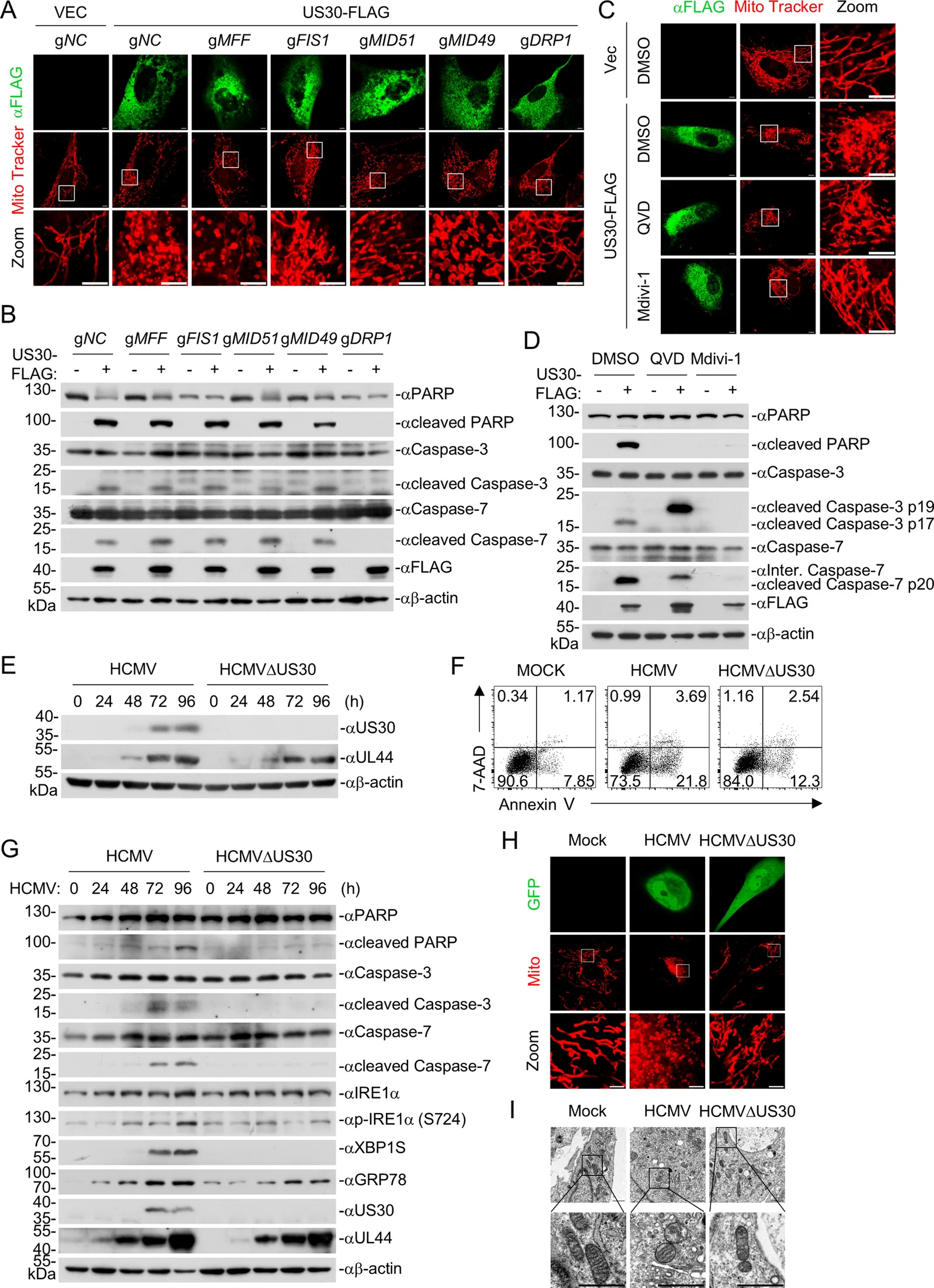
Roles of US30 in HCMV-induced UPR and apoptosis. (**A**) Effects of US30 on mitochondrial morphology in control and MFF-, FIS1-, MID51-, MID49-, and DRP1-deficient HFFs. HFFs (5 × 10^4^) edited with the indicated gRNA were transfected with US30-FLAG plasmid (0.2 μg) or empty vector for 24 h before confocal microscopy. Scale bars, 20 μm. (**B**) Effects of US30 on the cleavage of PARP and caspase-3/7 in control and the indicated gene-edited HEK293 cells. HEK293 cells (5 × 10^5^) edited with the indicated gRNA were transfected with US30-FLAG plasmid (0.5 μg) or empty vector for 24 h before immunoblotting analysis with the indicated antibodies. (**C**) Effects of QVD and Mdivi-1 on US30-induced mitochondrial fission. HFFs (5 × 10^4^) were transfected with US30-FLAG plasmid (0.2 μg) or empty vector for 4 h and then left untreated or treated with QVD (20 μM) or Mdivi-1 (50 μM) for 20 h before confocal microscopy. Scale bars, 20 μm. (**D**) Effects of QVD and Mdivi-1 on US30-induced cleavage of PARP and caspase-3/7. HEK293 cells (5 × 10^5^) were transfected with US30-FLAG plasmid (0.5 μg) or empty vector for 4 h and then left untreated or treated with QVD (20 μM) or Mdivi-1 (50 μM) for 20 h before immunoblotting analysis with the indicated antibodies. (**E**) Verification of US30-deficient HCMV. HFFs (5 × 10^5^) were infected with wild-type HCMV or HCMVΔUS30 (MOI = 1) for the indicated times. Cell lysates were subjected to immunoblotting analysis with the indicated antibodies. (**F**) US30 deficiency inhibits HCMV-induced apoptosis. HFFs (5 × 10^5^) were left uninfected or infected with wild-type HCMV or HCMVΔUS30 (MOI = 1) for 96 h before flow cytometry analysis of annexin V-PE/7-AAD stained cells. (**G**) Effects of US30 on HCMV-induced activation of UPR and apoptosis. HFFs (5 × 10^5^) were left uninfected or infected with wild-type HCMV or HCMVΔUS30 (MOI = 1) for the indicated times before immunoblotting analysis with the indicated antibodies. (**H**) Live-cell super-resolution imaging of mitochondria in HCMV-infected HFFs. HFFs (5 × 10^4^) were left uninfected or infected with wild-type HCMV or HCMVΔUS30 (MOI = 1) for 72 h and then stained with PK Mito Orange before live-cell super-resolution imaging analysis. Scale bars, 20 μm. (**I**) Transmission electron microscopy (TEM) of mitochondria in HCMV-infected HFFs. HFFs (5 × 10^6^) were left uninfected or infected with wild-type HCMV or HCMVΔUS30 (MOI = 1) for 72 h and then processed for TEM. Scale bars, 500 nm. All the experiments were repeated for at least two times with similar results.

Since US30 downregulates mitochondrial proteins (Fig. S1A and B), we next investigated whether this is related to US30-induced apoptosis. First, we examined the effects of US30 on mitochondrial functions. Flow cytometry analysis indicated that US30 increased the mean fluorescence intensity (MFI) of active mitochondria and reduced mitochondrial membrane potentials, suggesting that US30 perturbs mitochondrial homeostasis (Fig. S2A and B). Confocal microscopy indicated that ectopic expression of US30 caused marked fragmentation called fission of mitochondria (Fig. S2C), which has been shown to be mediated by DRP1 and its receptors including MFF, FIS1, MID51, and MID49 (23, 38). It has been reported that mitochondrial fission causes dysfunction of mitochondria and intrinsic apoptosis (39, 40). Therefore, we generated MFF-, FIS1-, MID51-, MID49-, and DRP1-deficient HFFs and HEK293 cells by the CRISPR-Cas9 method to determine whether US30-induced apoptosis is mediated by mitochondrial fission. The results indicated that DRP1-deficiency abrogated US30-induced mitochondrial fragmentation (Fig. 3A; Fig. S2D), and cleavage of PARP and caspase-3/7 (Fig. 3B; Fig. S2E), while deficiency of any one of the receptors had no marked effects (Fig. 3A and B), probably due to the redundant functions of these receptors in mitochondrial fission (41). Confocal microscopy indicated that Mdivi-1, a mitochondrial fission inhibitor, but not the caspase inhibitor QVD, inhibited US30-induced mitochondrial fragmentation (Fig. 3C; Fig. S2F). Additionally, both Mdivi-1 and QVD inhibited US30-induced cleavage of PARP and caspase-3/7 (Fig. 3D). These results collectively suggest that US30 induces DRP1-dependent mitochondrial fission and subsequent apoptosis.

### US30 deficiency inhibits HCMV-induced apoptosis and UPR

We next investigated the roles of viral US30 in the interaction of HCMV and host cells. To do this, we constructed US30-deficient (ΔUS30) and GFP-tagged HCMV (AD169 strain) following previously described procedures (42, 43). Given that the coding sequence of US30 partially overlaps with the coding sequence for ORF US29 at its 5′, we introduced a T-to-G mutation at nucleotide position 59 of US30-coding sequence to prematurely terminate US30 translation rather than deleting the US30-coding sequence to generate the HCMVΔUS30 strain (Fig. S3A). Immunoblotting experiments indicated that US30 was detected in HFFs infected with wild-type HCMV but not HCMVΔUS30 for 72 and 96 h (Fig. 3E), confirming the successful construction of US30-deficient HCMV. Additionally, deficiency of US30 had no significant effects on transcription of *IE1*, *IE2*, and *pp65* genes in the indicated times, indicating that the mutant virus exhibits similar replication kinetics compared to wild-type HCMV during the early phase of infection (Fig. S3B). We next determined the role of US30 in apoptosis induced by HCMV (AD169 strain), which has been previously reported (13). Flow cytometry analysis indicated that infection with HCMVΔUS30 caused less apoptosis compared to wild-type HCMV as shown by reduced population of annexin V-positive cells (Fig. 3F). Biochemical experiments indicated that infection of HCMV∆US30 caused reduced the activation of UPR and apoptosis compared to wild-type HCMV as characterized by decreased levels of p-IRE1α (S724), GRP78, and XBP1S and decreased cleavage of PARP and caspase-3/7 (Fig. 3G). Confocal microscopy indicated that HCMV infection induced mitochondrial fission at 24 hpi (Fig. S3C), consistent with previous reports (28, 44). Interestingly, mitochondrial branch length was further reduced at 72 hpi relative to 24 hpi, suggesting that late viral proteins contribute to enhanced mitochondrial fragmentation during HCMV infection (Fig. S3C). Live-cell super-resolution imaging (Fig. 3H; Fig. S3D) and transmission electron microscopy (TEM) (Fig. 3I) further confirmed that HCMV∆US30 had an impaired ability to induce mitochondrial fragmentation compared to wild-type HCMV. These results suggest that US30 is a critical mediator of HCMV-triggered induction of apoptosis and UPR.

### Identification of US30-associated proteins

To investigate the molecular mechanisms of US30-triggered UPR and apoptosis, we ectopically expressed FLAG-tagged US30 in HEK293 cells and performed proteomics analysis of US30-associated proteins. Among the top 10 candidate proteins, we successfully constructed 9 C-terminal HA-tagged expression clones (except for POTEJ) (Fig. S4A). Transient transfection and co-immunoprecipitation (co-IP) experiments indicated that US30 interacted with CALX, NDUFS2, FAF2, NPL4, CALR, TMED10, and NAP1L1 but not PSMC1 and MRPL54 (Fig. S4B).

### US30 impairs ERAD to activate NPL4-mediated UPR

**Fig 4:**
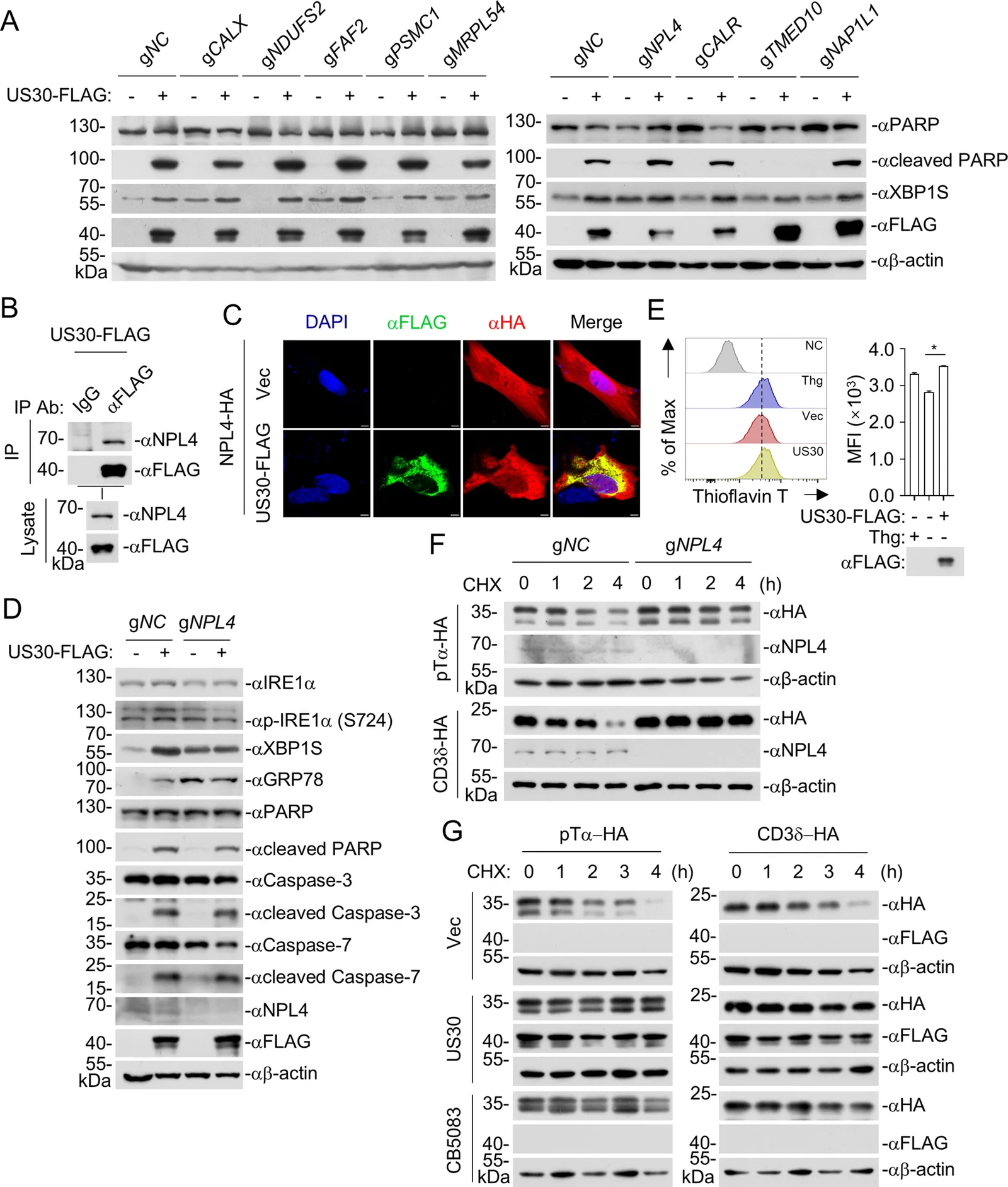
US30 impairs ERAD to induce UPR via NPL4. (**A**) Effects of the indicated proteins on US30-induced upregulation of cleaved PARP and XBP1S. HEK293 cells (5 × 10^5^) edited with the indicated gRNA were transfected with US30-FLAG plasmid (0.5 μg) or empty vector for 24 h before immunoblotting analysis with the indicated antibodies. (**B**) US30 is associated with endogenous NPL4. HEK293 cells (5 × 10^6^) were transfected with US30-FLAG plasmid (5 μg) for 24 h before semi-endogenous co-IP and immunoblotting analysis. (**C**) US30 is co-localized with NPL4 in the cytoplasm. HFFs (5 × 10^4^) were co-transfected with US30-FLAG (0.2 μg) and NPL4-HA (0.2 μg) plasmids for 24 h before confocal microscopy. Scale bars, 20 μm. (**D**) Effects of NPL4 on US30-induced UPR and apoptosis. The control and NPL4-deficient HEK293 cells (5 × 10^5^) were transfected with US30-FLAG plasmid (0.5 μg) or empty vector for 24 h before immunoblotting analysis with the indicated antibodies. (**E**) Effects of US30 on accumulation of aggregated proteins. HEK293 cells (5 × 10^5^) were transfected with US30-FLAG plasmid (0.5 μg) or empty vector for 18 h and then left untreated or treated with thapsigargin (Thg; 1 μM) for 6 h before flow cytometry analysis using thioflavin T staining. The levels of aggregated proteins are shown as representative fluorescence histograms (left) and the mean fluorescence intensity (MFI) (right). Data shown are mean ±  SEM from one representative experiment, *n*  =  3 independent samples. n.s., not significant. *P  <  0.05, **P  <  0.01. (**F**) NPL4-deficiency reduces pTα and CD3δ degradation. The control and NPL4-deficient HEK293 cells (5 × 10^5^) were transfected with pTα-HA or CD3δ-HA (0.2 μg) plasmids for 16 h and then treated with cycloheximide (CHX; 100 μg/mL) for the indicated times before immunoblotting analysis with the indicated antibodies. (**G**) US30 reduces pTα and CD3δ degradation. HEK293 cells (1 × 10^5^) were transfected with the indicated plasmids for 16 h following treatment with DMSO or CB5083 (10 μM) for 4 h and then treated with CHX (100 μg/mL) for the indicated times before immunoblotting analysis with the indicated antibodies. All the experiments were repeated for at least two times with similar results. n.s., not significant.

We next evaluated the potential roles of the US30-interacting proteins in US30-triggered UPR and apoptosis. As shown in Fig. 4A, knockout of NPL4, but not the other eight examined proteins, increased the basal level of XBP1S, and ectopic expression of US30 had no marked effects on the level of XBP1S in NPL4-deficient HEK293 cells. Notably, NPL4-deficiency had no marked effects on US30-induced cleavage of PARP in these experiments. These results suggest that NPL4 is a candidate mediator of US30-triggered UPR but not apoptosis.

NPL4 is an essential co-factor of the p97 ATPase complex in the ER, participating in the ERAD process via recognizing unfolded or misfolded proteins and delivering them to proteasomes for degradation (21). Inhibition of ERAD causes the accumulation of unfolded or misfolded protein aggregates, resulting in disrupted ER homeostasis and UPR (45). Semi-endogenous co-IP and confocal microscopy experiments confirmed that US30 was associated with NPL4 in the cytoplasm (Fig. 4B and C). Biochemical experiments indicated that NPL4-deficiency had no marked effects on US30-induced cleavage of PARP and caspase-3/7 (Fig. 4D). However, NPL4-deficiency significantly increased the basal levels of p-IRE1α, GRP78, and XBP1S, whereas US30 overexpression failed to further increase these UPR markers in NPL4-deficient cells, suggesting that NPL4 may function in the same signaling pathway as US30 and contribute to US30-mediated UPR activation but not apoptosis (Fig. 4D).

We next determined whether US30 activates UPR by disrupting NPL4-mediated ERAD and subsequently causing accumulation of protein aggregates. By staining cells with thioflavin T, a small molecule with fluorescence property and selectively binding to protein aggregates, we found that US30 induced increased accumulation of protein aggregates in HEK293 cells (Fig. 4E). We constructed two canonical ERAD substrates, pre-TCRα (pTα) and CD3δ, to examine their degradation rates in cycloheximide (CHX) assays (45, 46). The results indicated that knockout of NPL4 markedly reduced the degradation of pTα and CD3δ in CHX-treated cells, supporting a critical role of NPL4 in ERAD degradation (Fig. 4F). Additionally, ectopic expression of US30 impaired the degradation of pTα and CD3δ in HEK293 cells (Fig. 4G). In these experiments, CB5083, a potent inhibitor of p97 ATPase in the ERAD pathway (47), also inhibited the degradation of pTα and CD3δ (Fig. 4G). Collectively, these results suggest that US30 interacts with NPL4 to impair ERAD, leading to the accumulation of protein aggregates and the activation of UPR.

### US30 interacts with TMED10 to promote mitochondrial fission and apoptosis

**Fig 5:**
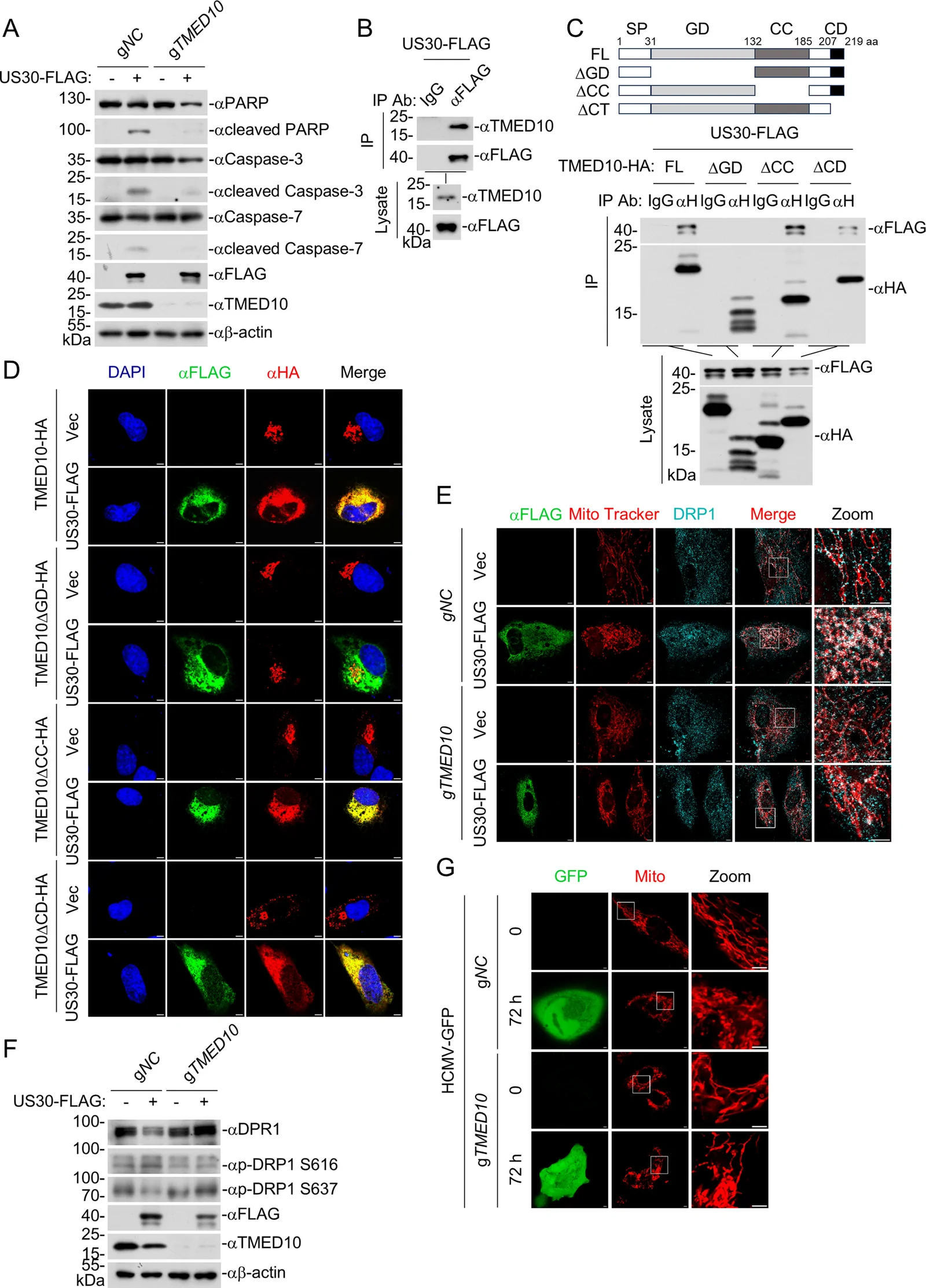
US30 interacts with TMED10 to promote mitochondrial fission. (**A**) Effects of TMED10 on US30-induced cleavage of PARP and caspase-3/7. The control and TMED10-deficient HEK293 cells (5 × 10^5^) were transfected with US30-FLAG plasmid (0.5 μg) or empty vector for 24 h before immunoblotting analysis with the indicated antibodies. (**B**) US30 is associated with endogenous TMED10. HEK293 cells (5 × 10^6^) were transfected with US30-FLAG plasmid (5 μg) for 24 h before semi-endogenous co-IP and immunoblotting analysis. (**C**) Association between US30 and TMED10 truncations. HEK293 cells (5 × 10^6^) were transfected with the indicated plasmids for 24 h before co-IP and immunoblotting analysis with the indicated antibodies. Schematic diagrams of TMED10 and its deletion mutants are shown. (**D**) Confocal microscopy of US30 and TMED10 truncations. HFFs (5 × 10^4^) were co-transfected with TMED10-HA truncations (0.2 μg) and US30-FLAG (0.2 μg) plasmids or empty vector for 24 h before confocal microscopy. Scale bars, 20 μm. (**E**) Effects of TMED10 on US30-induced mitochondrial fission and DRP1 recruitment to mitochondria. The control and TMED10-deficient HFFs (5 × 10^4^) were transfected with US30-FLAG plasmid (0.2 μg) or empty vector for 24 h before confocal microscopy. Scale bars, 20 μm. (**F**) Effects of TMED10 on US30-induced S616 phosphorylation and S637 dephosphorylation of DRP1. The control and TMED10-deficient HEK293 cells (5 × 10^5^) were transfected with US30-FLAG plasmid (0.5 μg) or empty vector for 24 h before immunoblotting analysis with the indicated antibodies. (**G**) Effects of TMED10 on HCMV-induced mitochondrial fission. The control and TMED10-deficient HFFs (5 × 10^4^) were left uninfected or infected with HCMV-GFP (MOI = 1) for 72 h before live-cell super-resolution imaging analysis. Scale bars, 20 μm. All the experiments were repeated for at least two times with similar results.

As shown above (Fig. 4A), knockout of TMED10 inhibited US30-triggered cleavage of PARP but not an increase of the XBP1S level, suggesting that TMED10 mediates US30-induced apoptosis but not UPR. This was further confirmed by the observation that TMED10-deficiency also inhibited US30-triggered cleavage of caspase-3/7 (Fig. 5A). It has been reported that TMED10 is mostly located in the ER-Golgi intermediate compartment (ERGIC) and involved in regulation of unconventional protein secretion (48). Semi-endogenous co-IP experiments indicated that ectopically expressed US30 was associated with endogenous TMED10 (Fig. 5B). TMED10 consists of an N-terminal signal peptide (SP), a luminal Golgi dynamics domain (GD), a middle coil-coil (CC) domain, and a C-terminal tail domain (CD) (48). Domain mapping experiments indicated that GD of TMED10 is responsible for its interaction with US30 (Fig. 5C). Confocal microscopy indicated that TMED10 was located at the ERGIC in HFFs, and ectopic expression of US30 caused redistribution of TMED10 to the cytoplasm, and at where they overlap (Fig. 5D). In these experiments, US30 induced redistribution of full-length TMED10, CC- or CT-truncated TMED10 mutants but failed to induce redistribution of the GD-truncated mutant (Fig. 5D), suggesting an important role of GD of TMED10 in mediating US30-induced effects.

Since our previous data have shown that US30 induced DRP1-dependent mitochondrial fission and subsequent apoptosis, we next investigated whether TMED10 mediates these processes. It has been reported that cytosolic DRP1 is activated via its S616 phosphorylation and S637 dephosphorylation and then recruited to mitochondrial outer-membrane to drive mitochondrial fission (26). Confocal microscopy indicated that TMED10-deficiency inhibited US30-induced recruitment of DRP1 to mitochondria and mitochondrial fragmentation in HFFs (Fig. 5E). Immunoblotting analysis indicated that ectopic expression of US30 upregulated the level of p-DRP1 (S616) and downregulated the level of p-DRP1 (S637) in HEK293 cells, and these effects were abrogated in TMED10-deficient cells (Fig. 5F). Live-cell super-resolution imaging experiments showed that TMED10-deficiency attenuated mitochondrial fragmentation caused by HCMV infection at 72 hpi (Fig. 5G; Fig. S5). These results collectively suggest that US30 interacts with TMED10, which alters the localization of TMED10 from ERGIC to the cytosol, where TMED10 promotes the activation of DRP1 and its translocation to mitochondria to induce mitochondrial fission and apoptosis.

### Domain mapping of US30

**Fig 6:**
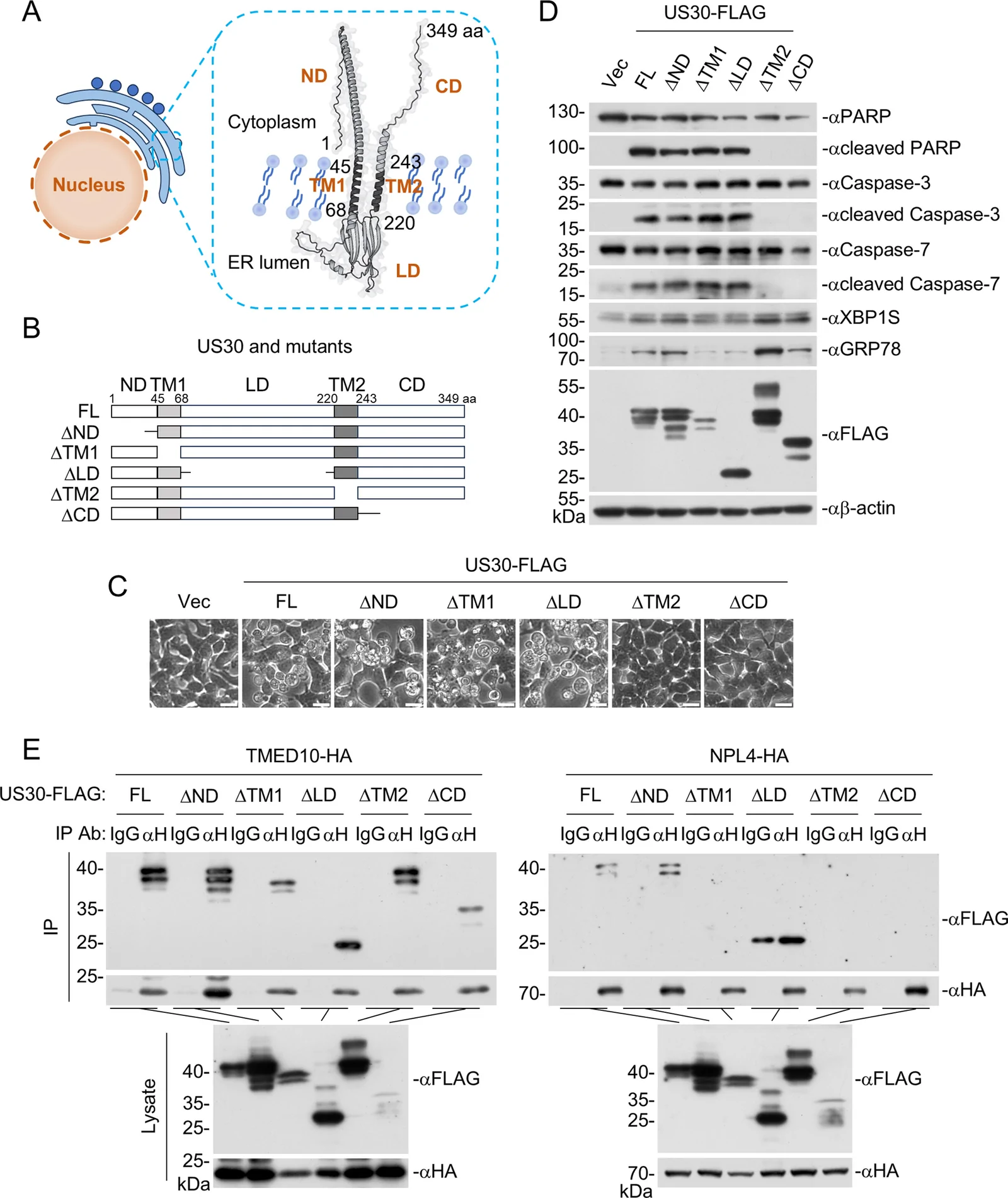
Domain mapping of US30. (**A**) Structure prediction of US30 using AlphaFold 3, TMHMM-2.0, and DeepLoc-2.0. (**B**) Schematic diagrams of US30 and its deletion mutants. (**C**) Effects of US30 truncations on cell death. HEK293 cells (5 × 10^5^) were transfected with US30-FLAG truncation plasmids (0.5 μg each) or empty vector for 24 h and then visualized by light microscopy. Scale bars, 20 μm. (**D**) Effects of US30 truncations on UPR and apoptosis. HEK293 cells (5 × 10^5^) were transfected with US30-FLAG truncation plasmids (0.5 μg each) or empty vector for 24 h before immunoblotting analysis with the indicated antibodies. (**E**) Interactions of US30 truncations with TMED10 or NPL4. HEK293 cells (5 ×  10^6^) were transfected with the indicated plasmids for 24 h before co-IP and immunoblotting analysis with the indicated antibodies. All the experiments were repeated for at least two times with similar results.

We next investigated the critical domains of US30 involved in activating apoptosis and UPR. Using a combination of AlphaFold 3, TMHHM 2.0 (predicting transmembrane proteins), and DeepLoc-2.0 (predicting protein subcellular localizations) programs, we identified US30 as a transmembrane protein with five domains, including a cytoplasmic N-terminal domain (ND), transmembrane domain 1 (TM1), luminal domain (LD), transmembrane domain 2 (TM2), and a cytoplasmic C-terminal domain (CD) (Fig. 6A). Based on this structural analysis, we constructed five truncations of US30 that lack each of the five domains (Fig. 6B). Transient transfection experiments indicated that the deletion of either TM2 or CD but not the other three domains of US30 abolished its ability to induce cell death (Fig. 6C) and the cleavage of PARP and caspase-3/7 (Fig. 6D), suggesting that TM2 and CD, but not the other domains of US30, are critical for its ability to trigger apoptosis. On the other hand, the deletion of either TM1 or LD, but not the other domains of US30, impaired its ability to induce upregulation of GRP78 and XBP1S (Fig. 6D), suggesting that TM1 and LD of US30 are required for its ability to induce UPR. These results further support our previous observations that US30-induced apoptosis and UPR are uncoupled. We next performed co-IP experiments to determine whether these identified domains of US30 differentially mediate the interaction of US30 with TMED10 or NPL4. The results indicated that the deletion of any one of the domains failed to abolish the interaction of US30 and TMED10, suggesting that US30 may interact with TMED10 via multiple domains (Fig. 6E). In addition, we found that TM1-, TM2-, or CD-deficient truncations of US30 failed to associate with NPL4, whereas the other mutants of US30 interacted with NPL4 (Fig. 6E), suggesting that proper location of CD of US30 in the cytosolic side, but not the other domains including ND and LD, is important for its interaction with cytosolic NPL4. Since the deletion of ND, TM2, or CD of US30 did not affect its ability to upregulate XBP1S and GRP78, while the deletion of TM1 or LD of US30 lost its ability to upregulate XBP1S and GRP78 (Fig. 6D), the simplest explanation for these observations is that US30 employs the cytoplasmic CD for its interaction with NPL4, the other domains of US30 are important for maintaining its proper membrane topology required for binding, and the LD of US30 may be responsible for blocking the binding of NPL4 to the unfolded or misfolded proteins in the ER, therefore blocking ERAD and inducing UPR. Collectively, these results suggest that US30 induces NPL4-mediated UPR and TMED10-mediated apoptosis via distinct domains.

### US30 promotes HCMV multiplication and dissemination via TMED10-DRP1 but not NPL4 axis

**Fig 7:**
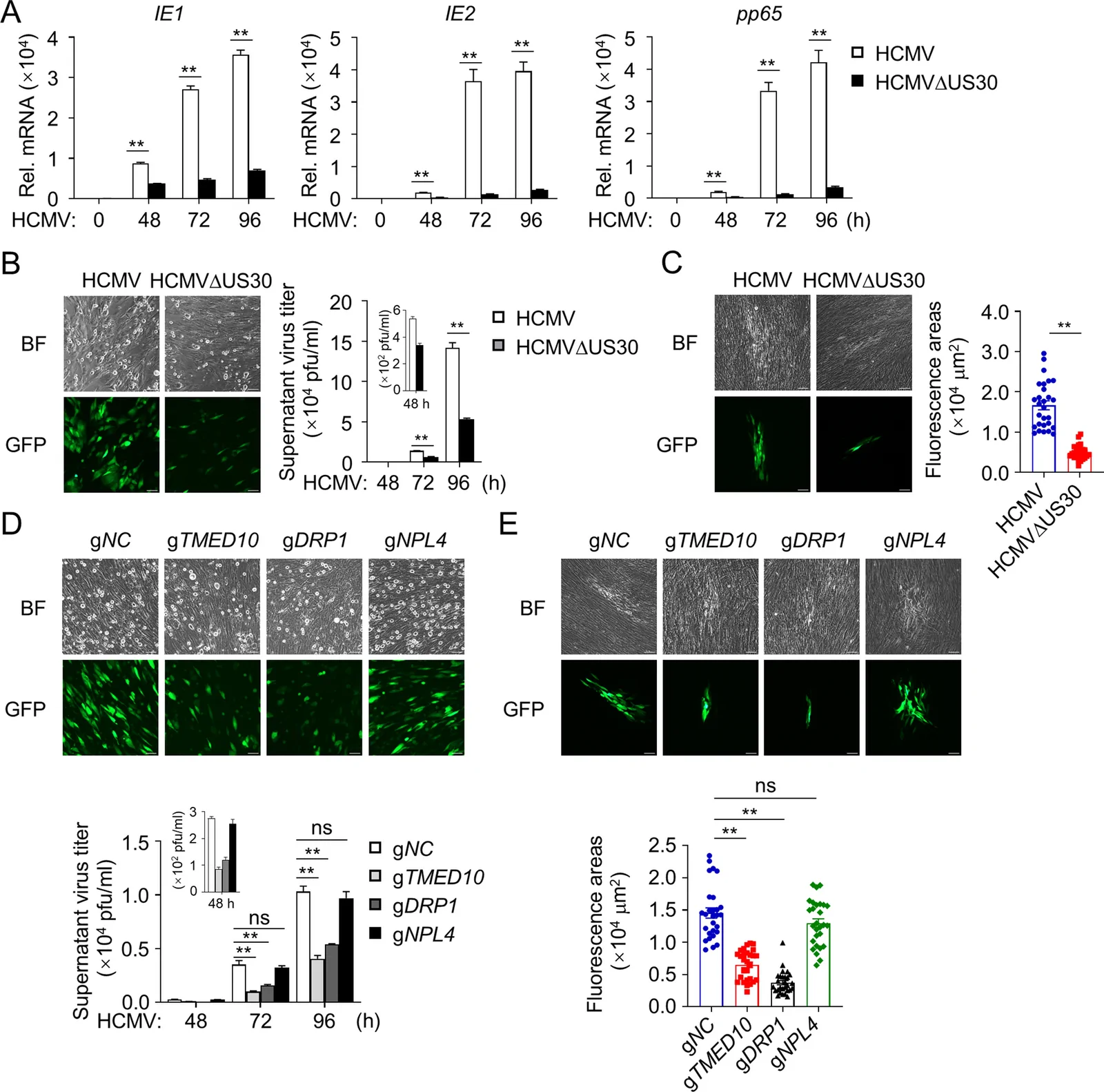
US30 drives HCMV multiplication and dissemination via TMED10-DRP1 axis but not NPL4. (**A**) Effects of US30-deficiency on HCMV-induced transcription of *IE1*, *IE2*, and *pp65*. HFFs (5 × 10^5^) were left uninfected or infected with wild-type HCMV or HCMVΔUS30 (MOI = 1) for the indicated times before qPCR analysis. (**B**) Effects of US30-deficiency on HCMV replication. HFFs (5 × 10^5^) were left uninfected or infected with wild-type HCMV or HCMVΔUS30 (MOI = 1) for the indicated times. The viral loads in the cells at 96 hpi were shown by fluorescent microscopy (left). Viral titers in the cell culture media were assessed by TCID_50_ (right). (**C**) Effects of US30-deficiency on HCMV dissemination. HFFs (5 × 10^6^) were infected with wild-type HCMV or HCMVΔUS30 (MOI = 0.1) for 14 days. Representative images of GFP-positive cells (left) and quantification of fluorescence areas (right) were shown. (**D**) Effects of TMED10-, DRP1-, or NPL4-deficiency on HCMV replication. HFFs (5 × 10^5^) were left uninfected or infected with HCMV (MOI = 1) for the indicated times. The viral loads in the cells at 96 hpi were shown by fluorescent microscopy (left). Supernatant virus titers at indicated times were assessed by TCID_50_ (right). (**E**) Effects of TMED10-, DRP1-, or NPL4-deficiency on HCMV dissemination. HFFs (5 × 10^6^) were infected with HCMV (MOI = 0.1) for 14 days. Representative images of GFP-positive cells (left) and quantification of fluorescence areas (right) were shown. The data shown are represented as mean ± SEM from one representative experiment (*n* = 3 independent samples for panels **A**, **B**, and **D**; *n* = 28 independent fluorescent areas in panels **C** and **E**). n.s., not significant. *P  <  0.05, **P  <  0.01.

Finally, we investigated the effects of US30-mediated apoptosis and UPR on HCMV life cycle. *IE1*, *IE2,* and *pp65* are canonical early genes of HCMV, which are used as markers for HCMV genome replication (7, 49). qPCR assays indicated that the deletion of US30 inhibited HCMV (MOI = 1)-induced transcription of *IE1*, *IE2*, and *pp65* in HFFs at 48, 72, and 96 hpi (Fig. 7A). Fluorescence microscopy and tissue culture infective dose 50% (TCID_50_) assays indicated that infection of HCMV∆US30 produced less progeny viruses both in the cells and culture medium compared to wild-type HCMV at 48, 72, and 96 hpi with MOI = 1 (Fig. 7B). We also quantified the sizes of spread areas formed by wild-type HCMV or HCMV∆US30 (MOI = 0.1) at 14 days post-infection (dpi) in HFFs. The spread areas of HCMVΔUS30 plaques, as characterized by GFP-positive areas, were significantly reduced compared to that of wild-type HCMV (Fig. 7C). These results suggest that US30 is important for HCMV replication and dissemination.

To address whether US30-induced mitochondrial fission/apoptosis and UPR are involved in regulation of HCMV life cycle, we determined the effects of deficiency of TMED10, DRP1, and NPL4 on HCMV life cycle in HFFs. The results indicated that deficiency of either TMED10 or DRP1, but not NPL4, suppressed the production of progeny viruses (Fig. 7D) and spreading to neighboring cells (Fig. 7E) of wild-type HCMV. These results suggest that US30 promotes HCMV replication and dissemination via TMED10-mediated mitochondrial fission and apoptosis but not NPL4-mediated UPR.

## DISCUSSION

HCMV and other herpesviruses have evolved to encode multiple proteins or microRNAs to favor viral replication and persistence via manipulating host cellular processes, including lysosomal degradation, ER proteostasis, and mitochondrial dynamics, which are related to cell fate (11, 12, 50, 51). To systematically investigate the effects of HCMV proteins on cell death, we performed expression screens of 118 HCMV-encoded clones and identified US30 as a viral factor that hijacks host mitochondrial fission and apoptosis to promote HCMV multiplication and dissemination.

**Fig 8:**
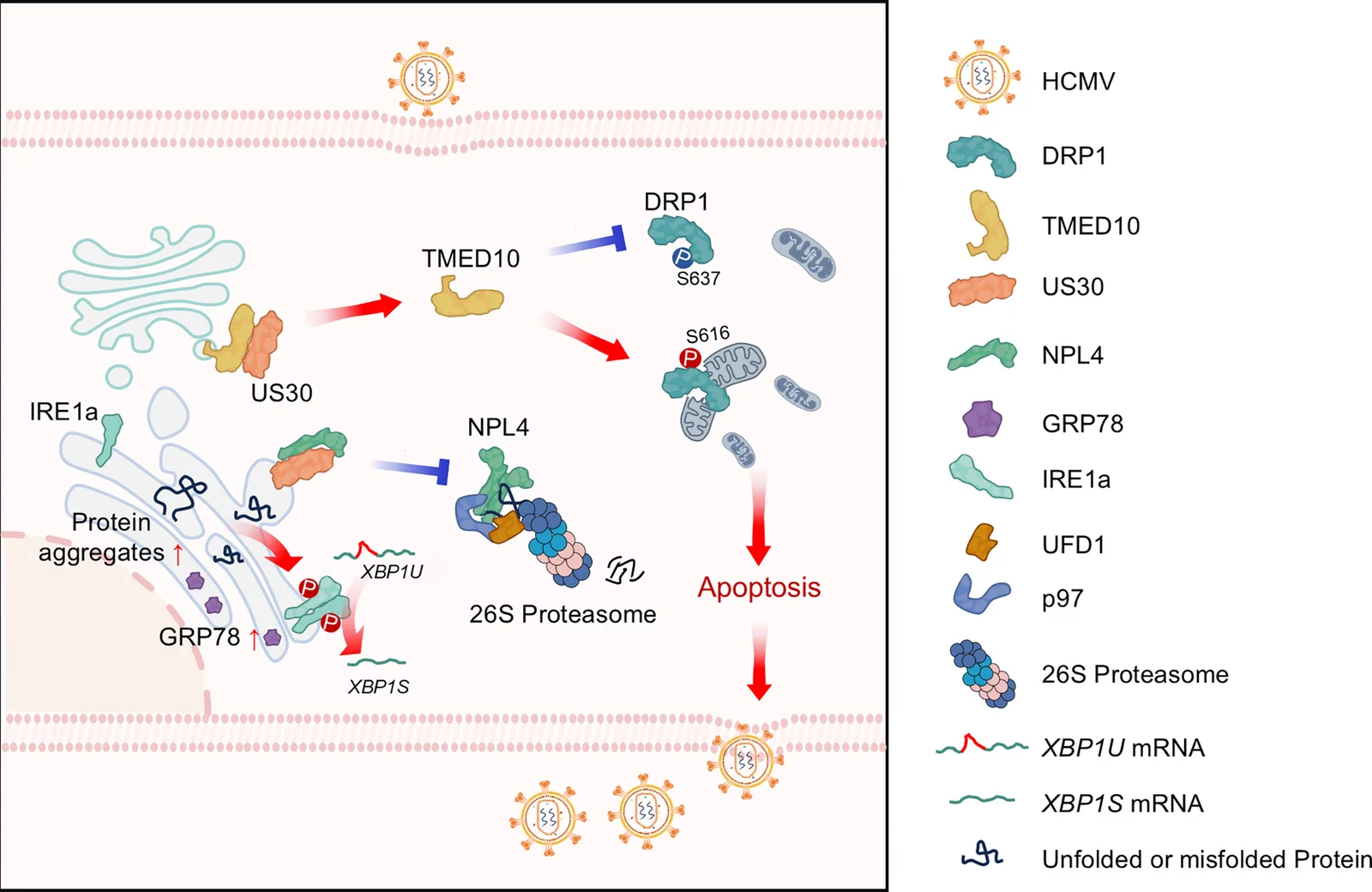
A working model for US30-mediated mitochondrial fission/apoptosis and UPR. See text for details.

Our results showed that ectopic expression of US30 induced caspase-dependent apoptosis, and US30-deficiency suppressed this process induced by infection of HCMV, suggesting a physiological role of US30 in mediating HCMV-induced apoptosis. We showed that US30 was located in both ER and mitochondria to disrupt their homeostasis as characterized by upregulation of UPR-related proteins in ER and downregulation of oxidative phosphorylation in mitochondria. Further experiments indicated that US30 activated IRE1α-mediated UPR and treatment with IRE1α endoribonuclease inhibitor (4μ8c) or an ER stress inhibitor (4-PBA) inhibited this process but failed to block US30-induced cell death, suggesting that US30-induced UPR and apoptosis are uncoupled (Fig. 8). In subsequent experiments, we found that US30 induced mitochondrial fragmentation/fission, which has been previously reported to trigger intrinsic apoptosis (39, 40). Our experiments revealed that deficiency of DRP1, a critical initiator of mitochondrial fission, or treatment with mitochondrial fission inhibitor Mdivi-1, inhibited US30-mediated mitochondrial fission, and cleavage of PARP and caspase-3/7, whereas treatment with the caspase inhibitor QVD inhibited US30-mediated apoptosis but had no marked effects on US30-induced mitochondrial fission. These results suggest that DRP1-mediated mitochondrial fission precedes apoptosis induced by US30. Previous studies have revealed that HCMV encodes several anti-apoptotic proteins during the early phase of infection, such as UL36 (vICA), which inhibits extrinsic apoptotic signaling to maintain HCMV infection (5). In contrast, our study identified US30 as a pro-apoptotic viral factor that promotes mitochondrial dysfunction during the late phase of infection. The opposing effects of these viral proteins imply that HCMV precisely modulates the survival-death balance at distinct stages of the viral life cycle.

By mass spectrometry analysis of US30-associated proteins, we identified TMED10, an ERGIC protein. Ectopic expression of US30 promoted the translocation of TMED10 from ERGIC to cytoplasm. Deficiency of TMED10 abrogated US30-induced S616 phosphorylation and S637 dephosphorylation of DRP1, a critical initiator of mitochondrial fission. Consistently, deficiency of TMED10 also impaired US30-mediated recruitment of DRP1 from the cytosol to mitochondria, as well as mitochondrial fission and subsequent apoptosis. These results suggest that the TMED10-DRP1 axis mediates US30-induced mitochondrial fission and apoptosis. Our identification of TMED10 as an upstream regulator of DRP1 activation reveals an alternative mechanism linking ERGIC-associated proteins to mitochondrial dynamics. This finding expands the current understanding of DRP1 regulation. Additionally, deficiency of TMED10 and DRP1 suppressed multiplication and dissemination of HCMV in HFF cells. These results suggest that US30-induced TMED10-DRP1-mediated mitochondrial fission and subsequent apoptosis contribute to HCMV multiplication and dissemination.

Although our data suggest that TMED10 is required for regulating US30-induced DRP1 phosphorylation, the molecular mechanisms by which TMED10 activates cytoplasmic DRP1 remain unclear. It has been reported that several kinases involve in the activation of DRP1 via regulating its S616 phosphorylation and S637 dephosphorylation, such as ROCK1, PKA, and CaMKII (25, 52, 53). It will be important for future studies to determine whether TMED10 modulates US30-induced activation of DRP1 via one or more of these kinases. In addition, how TMED10-mediated mitochondrial fission/apoptosis promotes HCMV multiplication and dissemination remains unclear. Previous reports have revealed that herpesviruses hijack cellular mitochondrial fission to promote viral replication via different strategies, such as inhibiting antiviral immunity and increasing host cell metabolism (6, 54, 55). Notably, although the early-phase HCMV proteins UL13 and UL37x1 (vMIA) have both been associated with mitochondrial fragmentation, these morphological changes are accompanied by distinct functional consequences (28, 56). UL13 promotes mitochondrial cristae remodeling and enhances oxidative phosphorylation, whereas pUL37x1 induces mitochondrial fragmentation while maintaining its anti-apoptotic activity by preventing mitochondrial outer membrane permeabilization. In contrast, our findings demonstrated that US30-mediated mitochondrial fragmentation is accompanied by mitochondrial dysfunction, characterized by loss of mitochondrial membrane potential and reduced mitochondrial oxidative phosphorylation, it would be interesting to investigate whether this is related to regulation of HCMV multiplication and dissemination.

Our experiments also indicated that US30 interacted with the p97 ATPase cofactor NPL4, and NPL4-deficiency inhibited US30-induced increase of IRE1α and XBP1S but had no marked effects on apoptosis, further confirming that US30-induced UPR and apoptosis are uncoupled. Consistent with a previous report that NPL4 acts as an essential compartment of p97 ATPase complex that is critical for ERAD (20), we found that NPL4-deficiency impaired the degradation of pTα and CD3δ, two canonical ERAD substrates (45, 46), and ectopic expression of US30 exhibited the similar inhibition on degradation of these ERAD substrates, suggesting that US30 activates NPL4-mediated UPR via disrupting ERAD and resulting in subsequent accumulation of unfolded or misfolded protein aggregates in ER. Domain mapping experiments indicated that TM1-, TM2-, or CD-deficient truncations of US30 failed to associate with NPL4, whereas the deletion of ND and LD of US30 retained its ability to interact with NPL4, suggesting that proper location of CD of US30 in the cytoplasmic side, but not the other domains including ND and LD, is important for its interaction with cytosolic NPL4. Since the deletion of ND, TM2, or CD of US30 did not affect its ability to induce UPR as indicated by upregulation of XBP1S and GRP78, while the deletion of TM1 or LD of US30 lost its ability to induce UPR, the simplest explanation for these observations is that US30 employs the cytoplasmic CD for its interaction with NPL4, the other domains of US30 are important for maintaining its proper membrane topology required for its binding to NPL4, and the LD of US30 may be responsible for blocking the binding of NPL4 to the unfolded or misfolded proteins in the ER, which is, therefore, required for blocking ERAD and inducing UPR. In our experiments, we found that NPL4-deficiency had no marked effects on HCMV multiplication and dissemination, suggesting that US30-NPL4-mediated UPR is not important for regulation of HCMV multiplication and dissemination *in vitro*. It would be interesting to investigate whether NPL4-mediated UPR plays a role in HCMV-host interaction *in vivo* when a proper mouse model is available in the future.

In conclusion, our findings identify HCMV US30 as a critical viral factor to activate both mitochondrial fission-mediated apoptosis and UPR in host cells. We also demonstrate that US30-mediated apoptosis contributes to the multiplication and dissemination of HCMV. This work expands our understanding of how HCMV integrates multiple host cellular pathways to control cell fate and regulate its life cycle and provides potential targets for intervention of HCMV-related diseases.

## MATERIALS AND METHODS

### Cells and cell culture

HFFs were kindly provided by Dr. Min-Hua Luo (Wuhan Institute of Virology, CAS). HEK293 cells were obtained from the American Type Culture Collection (ATCC). HFFs and HEK293 cells were cultured in Dulbecco’s modified Eagle’s medium (DMEM) (Gibco, C11995500BT) containing 10% fetal bovine serum (FBS) (Cell Max, SA211.02) at 37°C in a CO_2_ incubator.

### Viruses

HCMV AD169 strain tagged with GFP has previously been reported (57). Bacterial artificial chromosome pAD-GFP carrying the GFP-tagged genome of the HCMV AD169 strain (pAD-GFP-HCMV) was previously described (7, 42) and used to generate recombinant HCMV∆US30 virus. pAD-GFP-∆US30 BAC was generated by using a two-step recombination system in the GS1783 *Escherichia coli* strain as previously described (42). The following oligonucleotide primers were used to amplify the marker cassette linear fragment: Forward,CTCGATCGACGGCTACGGTTCAGGAATGTACCGTTCTGACCGCCCCGAATTGCGGCATCGTAGGGATAACAGGGTAATCGATTTA; Reverse, CGCCGTCGTTGTTGACGATGCCGCAATTCGGGGCGGTCAGAACGGTACATTCCTGAACCGCAACCAATTAACCAATTCTGATTAG.

The recombinant HCMV∆US30 virus was obtained by transfecting pAD-GFP-∆US30 BAC DNA into HFFs. The cell-free supernatant was collected when the entire monolayer of HFFs was lysed. The virus titer was determined by standard median tissue culture infective dose (TCID_50_) assays.

### Reagents and antibodies

CellTiter-Glo Luminescent Cell Viability Assays kit (Promega, G7573), Q-VD-Oph (MCE, HY-12305), ferrostatin-1 (MCE, HY-100579), ammonium tetrathiomolybdate (Target Mol, 15060-55-6), NH_4_Cl (Sinopharm Chemical Reagent Co., Ltd, 10001518), chloroquine (MCE, HY-17589A), 3-MA (MCE, HY-19312), olaparib (MCE, HY-10162), 4μ8c (MCE, HY-19707), 4-phenylbutyric acid (MCE, HY-A0281), thapsigargin (MCE, HY-13433), cycloheximide (Sigma, 239763), RNAiso plus (Takara Bio, 9109), SYBR Green Mix (Bio-Rad, 172-5274), M-MLV reverse transcriptase (Invitrogen, 28025-013), RiboLock RNase inhibitor (Thermo Scientific, EO0381), lipofectamine 2000 (Invitrogen, 52887), puromycin (Amresco, J593), and polybrene (Millipore, TR-1003-G) were purchased from the indicated companies.

Mouse antibodies against FLAG (Sigma, F3165, RRID:AB_259529), β-actin (Sigma, A2228, RRID:AB_476697), HA (BioLegend, 901515, RRID:AB_2565334), TMED10 (Santa, sc-137003, RRID:AB_2204336); rabbit antibodies against PARP (CST, 9542S, RRID:AB_2160739), cleaved PARP (CST, 5625S, RRID:AB_10699459), caspase-3 (CST, 9662S, RRID:AB_331439), cleaved caspase-3 (CST, 9664S, RRID:AB_2070042), caspase-7 (CST, 9492S, RRID:AB_2228313), cleaved caspase-7 (CST, 8438S, RRID:AB_11178377), calnexin (CST, 2433S, RRID:AB_2243887), SDHA (Proteintech, 14865-1-AP, RRID:AB_11182164), IRE1α (CST, 3294S, RRID:AB_823545), p-IRE1αS724 (Abclonal, AP1442, RRID:AB_3683584), GRP78 (Proteintech, 11587-1-AP, RRID:AB_2119855), XBP1S (CST, 40435S, RRID:AB_2891025), NPL4 (Proteintech, 11638-1-AP, RRID:AB_10597107), DRP1 (CST, 8570S, RRID:AB_10950498), p-DRP1 S616 (CST, 3455S, RRID:AB_2085352), p-DRP1 S637 (CST, 4867S, RRID:AB_10622027) were purchased from the indicated manufacturers. Antisera against US30 were generated by immunizing rabbits with purified recombinant US30 protein. Rabbit polyclonal antibody against UL44 was previously described (58).

### Constructs and transfection

The expression clones encoding 118 independent HCMV cDNAs were previously described (59, 60) and listed in Table S1. Expression plasmids for US30-FLAG and its mutants; NPL4-HA and its mutants; TMED10-HA and its mutants; pre-TCRα-HA and CD3δ-HA; and other C-terminal HA-tagged candidate clones were constructed by standard molecular biology techniques.

HEK293 cells were transfected by calcium phosphate precipitation or PEI. HFFs were transfected with lipofectamine 2000 according to procedures recommended by the manufacturer.

### RT-qPCR

RT-qPCR assays were previously described (61, 62). Briefly, total RNA was extracted from cells using RNAiso plus reagents, followed by reverse transcription to generate cDNA, which was quantitated by qPCR. Data shown are the relative abundance of the indicated mRNA normalized to that of β-actin. The qPCR primers were listed in Table S2.

### Subcellular fractionation

HEK293 cells (5 × 10^7^) were transfected with US30-FLAG plasmid or empty vector for 24 h. The cells were washed with PBS, digested by 0.05% trypsin, and centrifuged at 600 *g* for 5 min at 4°C. The cell pellet was resuspended in 10 mL of ice-cold isolation buffer A (225 mM mannitol, 75 mM sucrose, 0.1 mM EGTA, and 30 mM Tris-HCl, pH 7.4). The sample was homogenized using 10 mL syringes by mechanical shearing with 20 strokes and then centrifuged at 600 × *g* for 5 min at 4°C before collection of the supernatant and pellet (nuclei). The supernatant was centrifuged at 7,000 × *g* for 10 min at 4°C, and the supernatant (cytoplasmic fraction containing lysosomes and microsomes) and pellet (mitochondrial fraction) were gently resuspended in 10 mL of ice-cold isolation buffer without EGTA. The supernatant was further centrifuged at 20,000 × *g* for 30 min, and then the supernatant (containing cytosol and ER) and pellet (lysosomal fraction) were collected. This supernatant was centrifuged at 100,000 × *g* for 1 h, and then the supernatant (cytosol) and pellet (ER) were collected, respectively. The mitochondrial suspension was centrifuged at 10,000 × *g* for 10 min at 4°C, and then the supernatant was discarded, and the crude mitochondrial pellet was collected.

### Flow cytometry analysis

For apoptosis analysis, HEK293 cells (1 × 10^6^) were harvested, washed with PBS, and stained with annexin V-FITC/PI Apoptosis Kit (Elabscience, E-CK-A211); HFFs infected with HCMV-GFP were stained with annexin V (Biolegend, 640908, 6 μg/mL) and 7-AAD (Biolegend, 420404, 5 μg/mL). For labeling cellular compartments in live cells, single-cell suspensions of HEK293 cells were stained with Mito Tracker Red CMXRos (Invitrogen, M7512, 100 nM) or ER Tracker Red (Invitrogen, E34250, 500 nM) for 30 min at 37°C. Fluo-4, AM (Invitrogen, F14201, 2 μM), and TMRM (Invitrogen, I34361, 25 nM) were employed to characterize cytoplasmic Ca^2+^ and mitochondrial membrane potentials after staining for 20 min at 37°C. For detection of unfolded or misfolded proteins, HEK293 cells were collected to stain with thioflavin T (MCE, HY-D0218, 5 μM) for 30 min at 37°C. After staining, cells were resuspended in flow buffer (PBS with 1% FBS) and analyzed using a Cytoflex LX flow cytometer (Beckman). The data were analyzed by FlowJo (v10.6.2).

### CRISPR-Cas9 knockout

CRISPR-Cas9-mediated genome editing was described previously (63, 64). Briefly, double-stranded oligonucleotides corresponding to the target sequences were cloned into lenti CRISPR-V2 vector. Then, HEK293 cells were transfected with the indicated retroviral plasmids, along with packaging plasmids psPAX2 (Addgene, 12260) and pCMV-VSV-G (Addgene, 8454). Twelve hours post-transfection, the culture medium was replaced with fresh antibiotic-free medium. After an additional 48 h, the viral particles were harvested to infect HEK293 cells or HFFs in the presence of polybrene (8 μg/mL). The infected HEK293 cells or HFFs were subsequently selected with puromycin (2 μg/mL) for 7 days before experiments. The gRNA sequences were listed in Table S3.

### Confocal microscopy

HFFs were grown on 12-mm coverslips and transfected with the indicated plasmids or infected with HCMV for the indicated times. Cells were fixed with 4% paraformaldehyde for 10 min, followed by permeabilization with 0.1% Triton X-100 for 5 min. Subsequently, cells were blocked with PBS containing 3% bovine serum albumin (MP Biomedicals, 180728C) for 30 min at room temperature and then stained with the specified primary antibodies for 2 h and their corresponding secondary antibodies for 1 h. The nuclei were stained with DAPI for 10 min at room temperature and then washed with PBS for three times. The cells were mounted on slides for fluorescence microscopy. For live-cell super-resolution imaging, HFFs were uninfected or infected with HCMV for the indicated times and then stained with PK Mito Orange (Genvivo, PKMO-1, 250 nM) for 20 min without fixation. Images were captured with a fluorescent microscope (Abberior STEDYCON 100 × oil objective). Images were analyzed using ImageJ (Java 21.0.7).

### Co-immunoprecipitation and immunoblotting analysis

For transient transfection and co-immunoprecipitation experiments, transfected HEK293 cells were lysed in NP-40 lysis buffer containing 20 mM Tris-HCl (pH 7.4), 150 mM NaCl, 1 mM EDTA, 1% NP-40, 10 μg/mL aprotinin, 10 μg/mL leupeptin, and 1 mM phenylmethylsulfonylfluoride. For immunoprecipitation experiments, an aliquot of lysate was incubated with the indicated antibody or control IgG at 4°C for 3 h. The targeted proteins were isolated using protein G Sepharose beads (GE Healthcare). Immunoprecipitates were resolved by SDS-PAGE and analyzed by immunoblotting using the indicated antibodies.

### Transmission electron microscopy

HFFs were cultured in 60-mm petri dishes and left uninfected or infected with HCMV or HCMVΔUS30 (MOI = 1). Subsequently, cells were harvested at 72 hpi and then fixed with 3% (wt/vol) glutaraldehyde for 2 h at 4°C. After washing three times with 0.1 M PBS (pH 7.4), samples were post-fixed in 1% osmium tetroxide at room temperature for 2 h and washed three times with PBS. Subsequently, samples were dehydrated through a graded ethanol series (50%, 70%, 80%, 90%, 95%, and 100%; 15 min each) and then infiltrated with a 1:1 mixture of acetone and epoxy resin (SPI), followed by pure resin infiltration. Embedding was carried out at 60°C for 48 h. Ultrathin sections (60–80 nm) were prepared using an ultramicrotome (Leica UC7, Leica, Germany) equipped with a diamond knife (Ultra 45°, Diatome, Switzerland). Sections were mounted on grids and stained with uranyl acetate (saturated solution in 2% ethanol, 15 min) and lead citrate. Finally, sections were examined using a transmission electron microscope (H-1400, Hitachi, Japan), and images were subsequently processed by ImageJ (Java 21.0.7).

### IP/MS

HEK293 cells were transfected with an empty vector or US30-FLAG plasmid for 24 h. Following transfection, cells were harvested and lysed in 1 mL NP-40 lysis buffer (20 mM Tris-HCl [pH 7.4], 150 mM NaCl, 1 mM EDTA, 1% NP-40, 10 μg/mL aprotinin, 10 μg/mL leupeptin, and 1 mM phenylmethylsulfonyl fluoride). Subsequently, the samples were centrifuged at 13,000 rpm for 10 min at 4°C, and then the supernatants were subjected to incubation with anti-FLAG affinity gel for 3 h at 4°C. The immunoprecipitated fractions were subsequently washed three times with washing buffer (NP-40 lysis buffer containing 750 mM NaCl) followed by three additional washes with PBS. The samples without the supernatants were analyzed by mass spectrometry (MS). MS raw data were processed using MaxQuant (v2.1.2.0). Label-free protein quantification was performed using the MaxLFQ algorithm. Search results were filtered with 1% false discovery rate (FDR) at both peptide and protein levels.

### Proteomics analysis

HEK293 cells were transfected with empty vector or US30-FLAG plasmid for 24 h. Following transfection, cells were harvested for proteomics analysis. DIA raw mass spectrometry data were processed using DIA-NN (v1.9) in library mode. Spectral files were searched against the UniProt human protein database. Precursor-level FDR was controlled at 1% to ensure reliable peptide identifications. Protein intensities were normalized using the MaxLFQ algorithm for quantitative analysis.

### TCID_50_ assays

HFFs were seeded in 96-well plates overnight prior to TCID_50_ assay. HCMV stocks or supernatants were serially diluted 1:10 to 1:10⁶ in DMEM. HFFs were incubated with 100 μL of each diluted virus for 2 h and then replaced with 200 μL of DMEM containing 2% FBS to maintain cell growth for 7 days. Viral titers were calculated using the Reed-Muench method.

### Statistical analysis

All data were processed and analyzed using GraphPad Prism 10. Statistical tests were selected according to the experimental design. For comparisons between two independent groups, an unpaired two-tailed Student’s *t*-test was performed. For comparisons among multiple groups, one-way analysis of variance (one-way ANOVA) followed by Tukey’s multiple comparison test was used. For comparisons involving more than two groups under the influence of two categorical variables, a two-way analysis of variance (two-way ANOVA) followed by Sidak’s multiple comparison test was performed. All quantitative data in histograms were presented as means ± standard error of the mean (SEM). *P* value < 0.05 were considered statistically significant. The number of asterisks indicates the degree of significance, with statistical significance set as *P < 0.05 and **P < 0.01.

## Data Availability

All study data are included in the article and/or supplemental material. The mass spectrometry proteomics data have been deposited to the ProteomeXchange Consortium under accession number PXD077060 and are publicly available through iProX (https://www.iprox.cn//page/project.html?id=IPX0016565000).

## SUPPLEMENTAL MATERIAL

Supplemental material mbio.01740-26-s0001.pdf Fig. S1–S5; Tables S1–S3.

ASM does not own the copyrights to Supplemental Material that may be linked to, or accessed through, an article. The authors have granted ASM a non-exclusive, world-wide license to publish the Supplemental Material files. Please contact the corresponding author directly for reuse.
