# Supplementary material for: HCMV US30 induces TMED10-DRP1-mediated mitochondrial fission and apoptosis to promote viral multiplication
Source: mBio. 2026 Sep 22;17(10):e01740-26. doi: 10.1128/mbio.01740-26 (PMC13730045; doi:10.1128/mbio.01740-26)
Supplement: Supplementary file 1 [file mbio.01740-26-s0001.pdf]

Figure S1

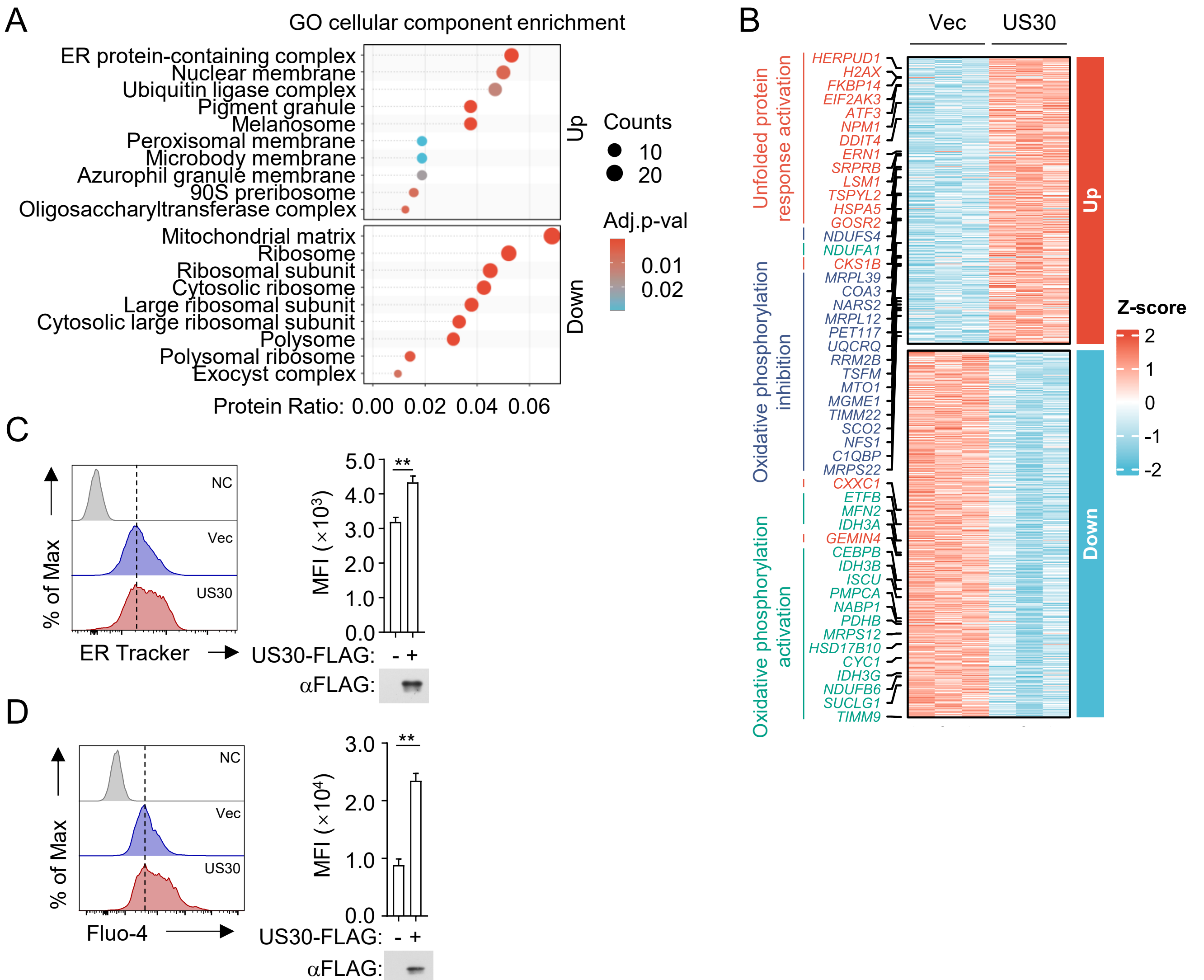

Figure S1. US30 disrupts ER homeostasis and mitochondrial oxidative phosphorylation

(A) Gene Ontology Cellular Component (GO:CC) enrichment analysis of the up-regulated and down-regulated proteins with a significance threshold set at $P < 0.05$. HEK293 cells (2×10^7) were transfected with US30-FLAG plasmid (5 μg) or empty vector for 24 h before proteomic analysis. Top 10 enriched terms were shown.

(B) Heatmap of the differentially expressed proteins in US30- versus empty vector-transfected HEK293 cells. Red labels indicate proteins that induce UPR, green labels indicate proteins that promote oxidative phosphorylation, and blue labels indicate proteins that inhibit oxidative phosphorylation.

(C) US30 increases ER signal. HEK293 cells (5×10^5) were transfected with US30-FLAG plasmid (0.5 μg) or empty vector for 24 h and analyzed by flow cytometry using ER Tracker red. The ER signal is shown as representative fluorescence histogram (left) and mean fluorescence intensity (MFI) (right).

(D) US30 increases the level of cytosolic Ca^{2+}. HEK293 cells (5×10^5) were transfected with US30-FLAG plasmid (0.5 μg) or empty vector for 24 h before analyzed by flow cytometry using Fluo-4. Cytosolic Ca^{2+} levels are shown as representative fluorescence histograms (left) and mean fluorescence intensity (MFI) (right).

The data shown in C and D are represented as mean ± SEM from one representative experiment, $n = 3$ independent samples. C and D were repeated for at least two times with similar results. n.s., not significant. * $P < 0.05$, ** $P < 0.01$.

Figure S2

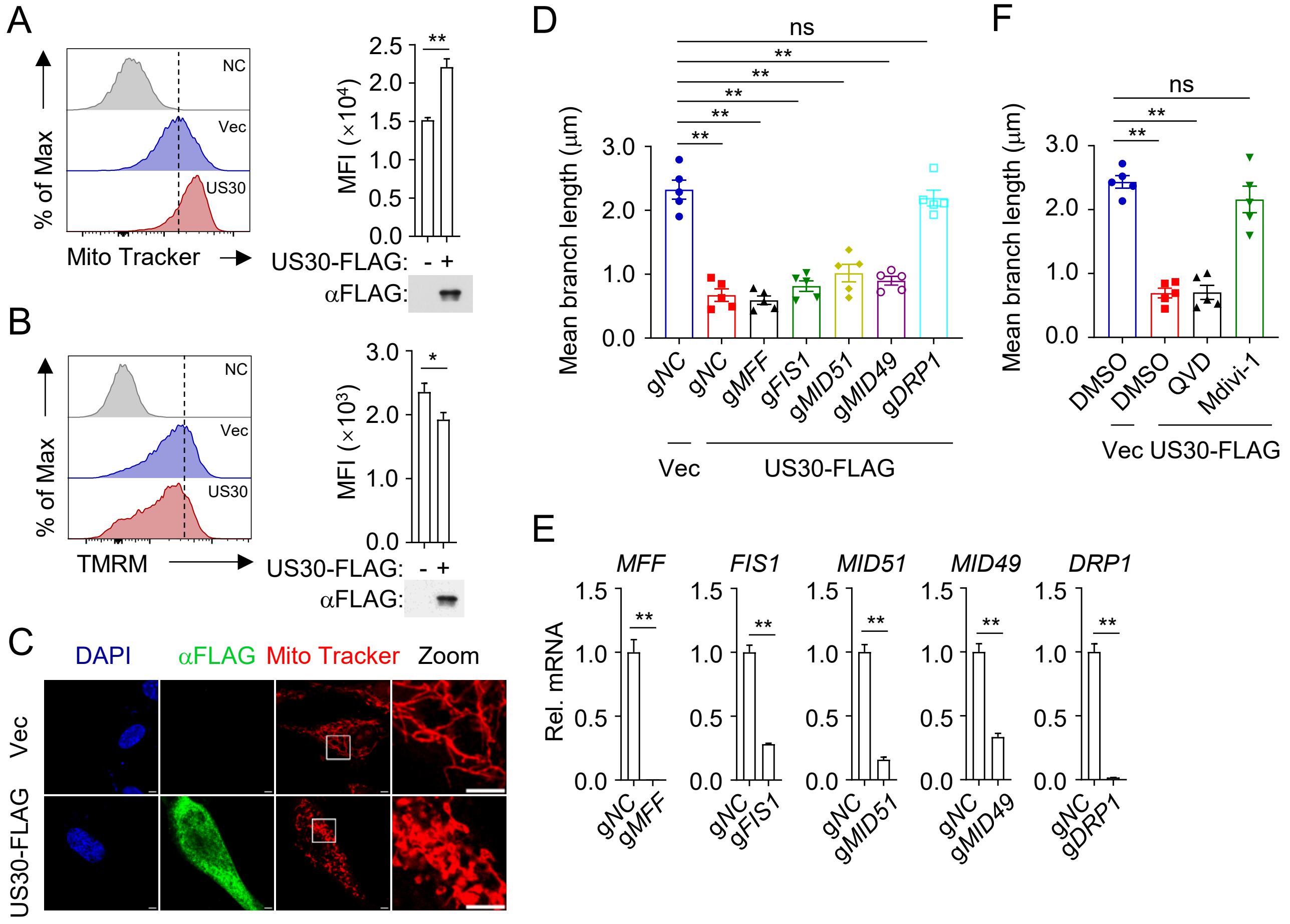

Figure S2. US30 disrupts mitochondrial homeostasis

(A) US30 increases mitochondrial signal. HEK293 cells (5×10^5) were transfected with US30-FLAG plasmid (0.5 µg) or empty vector for 24 h before analyzed by flow cytometry using Mito Tracker red. The mitochondrial signal is shown as representative fluorescence histogram (left) and mean fluorescence intensity (MFI) (right).

(B) US30 impairs mitochondrial membrane potentials. HEK293 cells (5×10^5) were transfected with US30-FLAG plasmid (0.5 µg) or empty vector for 24 h before analyzed by flow cytometry using TMRM. Mitochondrial membrane potential level is shown as representative fluorescence histogram (left) and mean fluorescence intensity (MFI) (right).

(C) Effects of US30 on mitochondrial morphology in HFFs. HFFs (5×10^4) were transfected with US30-FLAG plasmid (0.2 µg) or empty vector for 24 h before confocal microscopy. Scale bars, 20 µm.

(D) Effects of US30 on mitochondrial morphology in control and MFF-, FIS1-, MID51-, MID49-, and DRP1-deficient HFFs. Quantitative analysis of mitochondrial branch length was performed by ImageJ (n = 5 independent samples).

(E) Knockout efficiency of MFF-, FIS1-, MID51-, MID49-, and DRP1-deficient HEK293 cells. The control and MFF-, FIS1-, MID51-, MID49-, and DRP1-deficient HEK293 cells (5×10^5) were collected for qPCR analysis.

(F) Effects of QVD and Mdivi-1 on US30-induced mitochondrial fission. Quantitative analysis of mitochondrial branch length was performed by ImageJ (n = 5 independent samples).

The data shown in A, B, D, E and F are represented as mean ± SEM from one representative experiment, n = 3 independent samples in A, B and D. All the experiments were repeated for at least two times with similar results. n.s., not significant. * $P < 0.05$, ** $P < 0.01$.

Figure S3

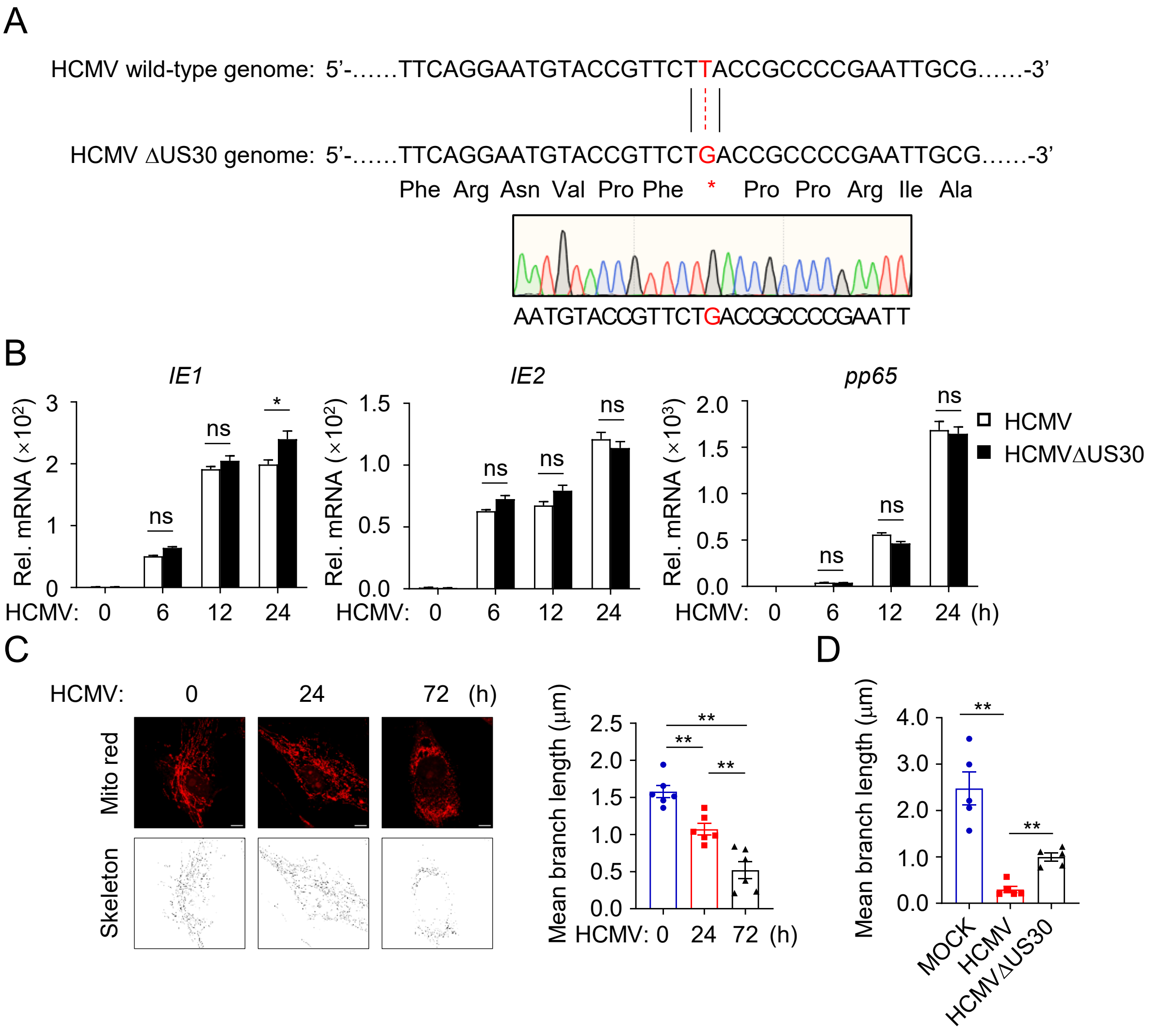

Figure S3. Construction of US30-deficient HCMV and effects of wild-type HCMV on mitochondrial morphology

(A) A scheme for homologous recombination-mediated genome editing of the US30 gene locus.

(B) Effects of US30-deficiency on HCMV-induced transcription of *IE1*, *IE2*, and *pp65*. HFFs (5×10^5) were left uninfected or infected with wild-type HCMV or HCMVΔUS30 (MOI = 1) for the indicated times before qPCR analysis (n = 3 independent samples).

(C) Effects of HCMV on mitochondrial morphology in HFFs. HFFs (5×10^4) were left uninfected or infected with wild-type HCMV (MOI = 1) for the indicated times and then stained with Mito Tracker red. ImageJ was used to skeletonize mitochondria and quantitative analysis of mitochondrial branch length was presented on the right (n = 6 independent samples). Scale bars, 20 μm.

(D) Effects of US30 on mitochondrial morphology during HCMV infection. Quantitative analysis of mitochondrial branch length in HCMV-infected HFFs (n = 5 independent samples).

The data shown in B, C, and D are represented as mean ± SEM from one representative experiment. n.s., not significant. * $P < 0.05$, ** $P < 0.01$.

Figure S4

A

Rank	Protein	Description
1	CALX	Calcium-binding protein
2	NDUFS2	NADH dehydrogenase iron-sulfur protein 2
3	POTEJ	POTE ankyrin domain family member J
4	FAF2	FAS-associated factor 2
5	PSMC1	26S proteasome regulatory subunit 4
6	MRPL54	Large ribosomal subunit protein mL54
7	NPL4	Nuclear protein localization protein 4 homolog
8	CALR	Calcium-binding chaperone
9	TMED10	Transmembrane emp24 domain-containing protein 10
10	NAP1L1	Nucleosome assembly protein 1-like 1

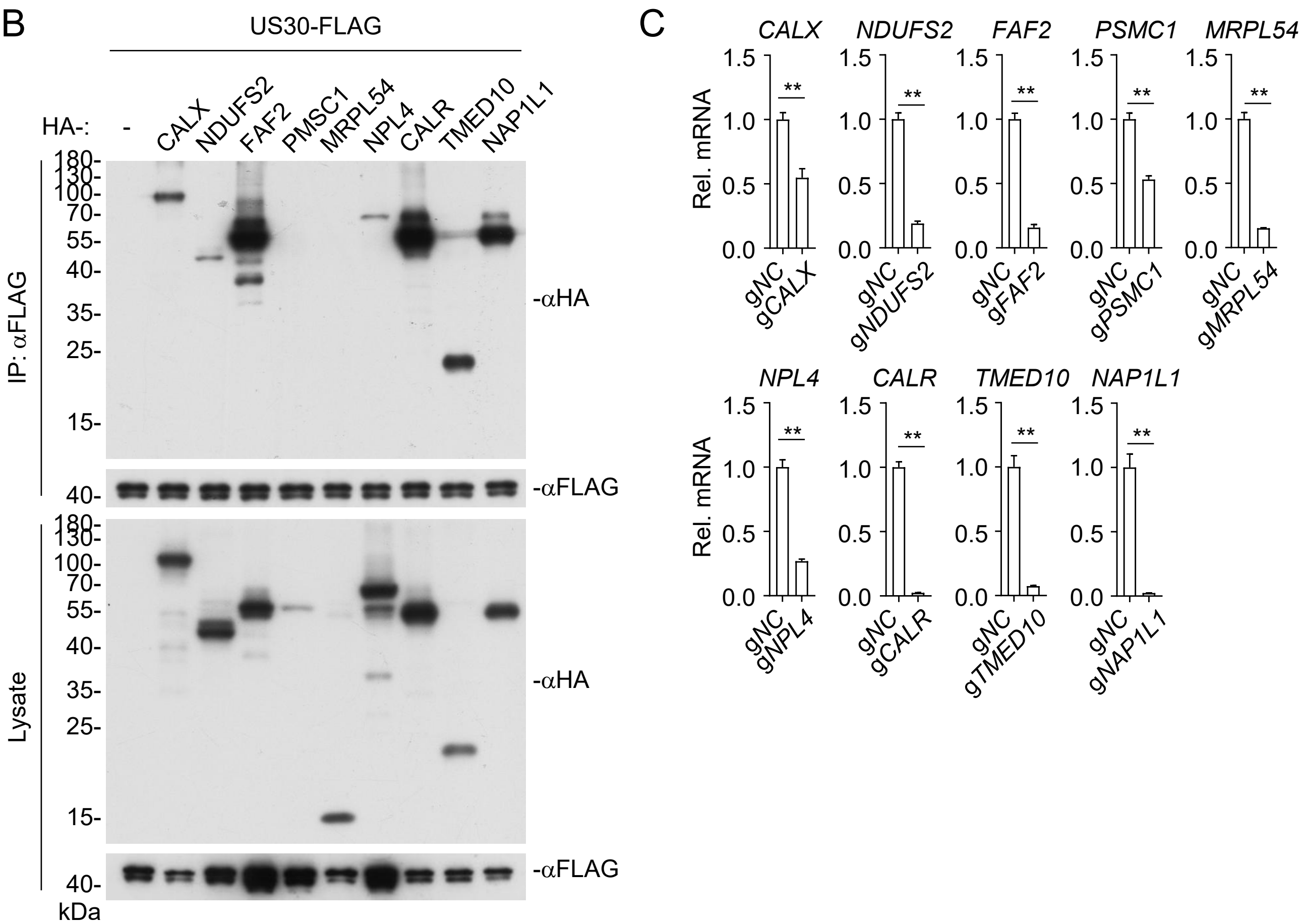

Figure S4. Identification of US30-associated proteins

(A) A list of top 10 candidate US30-associated proteins. HEK293 cells (2×10^7) were transfected with US30-FLAG plasmid (5 μg) or empty vector for 24 h before IP/MS analysis.

(B) Association of US30 with the candidate proteins. HEK293 cells (5×10^6) were transfected with US30-FLAG plasmid (5 μg) and the indicated candidate clones for 24 h before co-IP and immunoblotting analysis.

(C) Knockout efficiency of the candidate proteins in HEK293 cells. Control and the indicated gene-edited HEK293 cells (5×10^5) were collected for qPCR analysis.

Figure S5

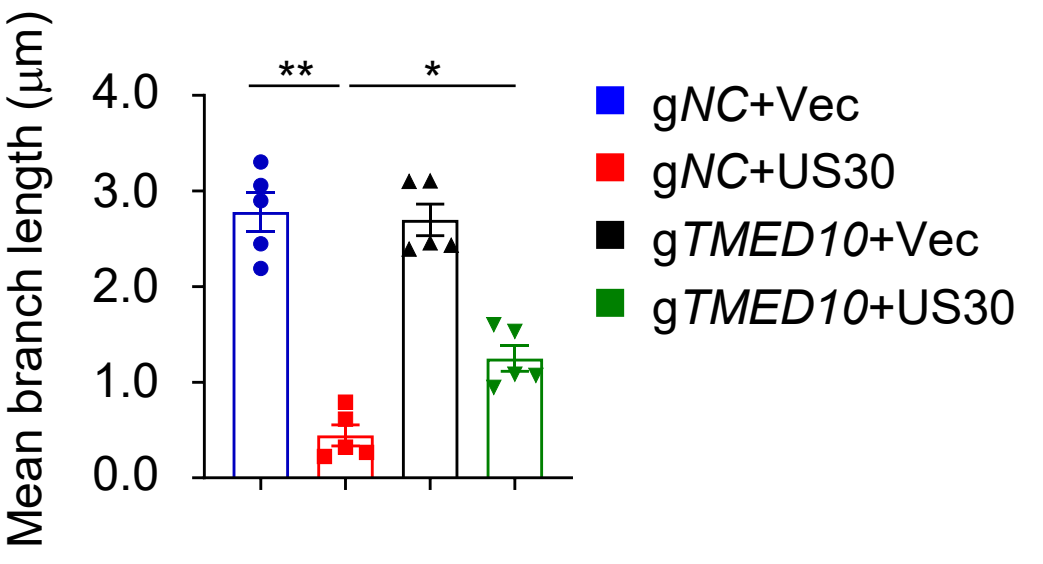

Figure S5. TMED10-deficiency attenuated HCMV-induced mitochondrial fission

Quantitative analysis of mitochondrial branch length in HCMV-infected HFFs. Data shown is represented as mean ± SEM from one representative experiment, (n = 5 independent samples). n.s., not significant. * $P < 0.05$, ** $P < 0.01$.

Table S1

Table S1. 118 expression clones encoding HCMV (AD169 strain) proteins.

HCMV-encoded proteins							
Number	Protein name	Number	Protein name	Number	Protein name	Number	Protein name
1	UL2	31	UL98	61	UL5	91	UL91
2	UL4	32	UL100	62	UL7	92	UL93
3	UL6	33	UL114	63	UL9	93	UL95
4	UL10	34	UL116	64	UL11	94	UL97
5	UL16	35	UL120	65	UL13	95	UL99
6	UL18	36	UL124	66	UL15A	96	UL103
7	UL20	37	UL128	67	UL17	97	UL105
8	UL22A	38	UL130	68	UL19	98	UL111A
9	UL24	39	UL132	69	UL21A	99	UL115
10	UL30A	40	UL148B	70	UL23	100	UL117
11	UL30	41	UL150A	71	UL25	101	UL119
12	UL34	42	US2	72	UL27	102	UL121
13	UL38	43	US6	73	UL31	103	UL123
14	UL40	44	US8	74	UL33	104	UL145
15	UL42	45	US10	75	UL35	105	US1
16	UL44	46	US12	76	UL43	106	US3
17	UL46	47	US14	77	UL45	107	US7
18	UL48A	48	US16	78	UL49	108	US9
19	UL50	49	US18	79	UL51	109	US11
20	UL72	50	US20	80	UL53	110	US13
21	UL74	51	US22	81	UL55	111	US15
22	UL74A	52	US28	82	UL57	112	US17
23	UL76	53	US30	83	UL69	113	US19
24	UL80	54	US32	84	UL71	114	US21
25	UL80.5	55	US34	85	UL73	115	US23
26	UL82	56	US34A	86	UL75	116	US29
27	UL84	57	RL1	87	UL77	117	US31
28	UL92	58	RL9A	88	UL79	118	US33A
29	UL94	59	RL11	89	UL83		
30	UL96	60	UL1	90	UL85		

Table S2

Table S2. qPCR sequences used in this study.

Gene name	Primer forward	Primer reverse
Human *ACTB*	CACCATTGGCAATGAGCGGTTC	AGGTCTTTGCGGATGTCCACGT
Human *CHOP*	GGAAACAGAGTGGTCATTCCC	CTGCTTGAGCCGTTCATTCTC
Human *GADD34*	ATGATGGCATGTATGGTGAGC	AACCTTGCAGTGTCCTTATCAG
Human *XBP1U*	CAGCTCAGACTGCCAGAGATCG	GTGCACGTAGTCTGAGTGCTGC
Human *XBP1S*	CCCCGAGGAGAAGGCGCTGAGG	TGCACCTGCTGCGGACTCAGCA
Human *MFF*	CTTTCACTCAGCGTAAGTACACGAG	GATAATGCAAGTTCCGGAGAGGATT
Human *FIS1*	TACGAGAAGGCCTTAAAGTACGTCC	CACACCCAGGGCCATGCCTCCCACG
Human *MID51*	CCATTGACTTTGTGCTCTCCAATGC	TTTTCCCCGAATGGCTCAGGCGGGT
Human *MID49*	AGCCAGCTGTTTGGCCAAAGCCACC	GAAGGGCCTGCAGAAACTGATCCTG
Human *DRP1*	TCTTCTAAATAGCTACGGTGAACCC	CAAATTCTAGCACCACCGCATAGCT
Human *CANX*	CTTTGTAAGTAACCTTGGGAGATGA	GTTGCTGTGTATGTTACTGGTGCTT
Human *NDUFS2*	CCCCACTCAATTCCATCACTAGTCG	CAAGCAAAGAAACAGCCCACTGGAA
Human *FAF2*	CAGTAATCCACGACTTCTTATTCTC	CTGTGTGGCTGAGTCCGGCCTCCTG
Human *PSMC1*	GGGGACCCCGATGTCAGTAGGAACC	TTGAGCAGGACCGAGCAGCCAGGTT
Human *MRPL54*	CCATGGGCGTCAACATCTACAAGGA	GCAGCCGCCGCCAGTACTCCCGGCT
Human *NPL4*	TGAGATTGATCAGTACCTCAGCAAA	GGAGGCTCGAGATGGTTTAGATAGT
Human *CALR*	CAGTTCCGGCAAGTTCTACGGTGAC	TGCTCATGTTTCACCGTGAACTGCA
Human *TMED10*	CAGCAGAATCTGTGATCTTGAGGTG	CCATCTCCTTCCATCTGCCCATTAA
Human *NAP1L1*	CTTGATGGTCTGGTAGAAACACCAA	CTTCCTTTCAAGATCATGAACTTCC
HCMV *US30*	GCTACGGTTCAGGAATGT	TCCAACATCGACGGTGCA
HCMV *IE1*	CAAGTGACCGAGGATTGCAA	CACCATGTCCACTCGAACCTT
HCMV *IE2*	ATGAACCACCCTCCTCTTCC	GATATTGCGCACCTTCTCGT
HCMV *pp65*	TGGAGAACGTGTCGGTCAAC	GGATGTTCAGCATCTTGAGCG

Table S3

Table S3. gRNA sequences used in this study.

Primer name	Primer forward	Primer reverse
g*NC*	CACCGGTAGTCGGTACGTGACTCGT	AAACACGAGTCACGTACCGACTACC
g*NDUFS2*-1	CACCGTCAATTCCATCACTAGTCGC	AAACGCGACTAGTGATGGAATTGAC
g*NDUFS2*-2	CACCGGTGTTGGGGCCCAAAGTTCA	AAACTGAACTTTGGGCCCCAACACC
g*MRPL54*-1	CACCGGCGTCAACATCTACAAGGAA	AAACTTCCTTGTAGATGTTGACGCC
g*MRPL54*-2	CACCGTGGCGACCAAACGCCTTTTC	AAACGAAAAGGCGTTTGGTCGCCAC
g*NPLOC4*-1	CACCGGTACTTATACGGACGGTACA	AAACTGTACCGTCCGTATAAGTACC
g*NPLOC4*-2	CACCGAGATGCCATTCGGCCACGGG	AAACCCCGTGGCCGAATGGCATCTC
g*CANX-1*	CACCGGCTTGGAACTGCTATTGTTG	AAACCAACAATAGCAGTTCCAAGCC
g*CANX-2*	CACCGGTGGTTGCTGTGTATGTTAC	AAACGTAACATACACAGCAACCACC
g*TMED10*-1	CACCGTAACGGAAAAGGGCCGCGCC	AAACGGCGCGGCCCTTTTCCGTTAC
g*TMED10*-2	CACCGGAGGCACTTGCGAGAGTTAA	AAACTTAACTCTCGCAAGTGCCTCC
g*CALR-1*	CACCGCGGCAAGTTCTACGGTGACG	AAACCGTCACCGTAGAACTTGCCGC
g*CALR-2*	CACCGTGCACGCTTTTATGCTCTGT	AAACACAGAGCATAAAAGCGTGCAC
g*NAP1L1*-1	CACCGGGTCTGGTAGAAACACCAAC	AAACGTTGGTGTTTCTACCAGACCC
g*NAP1L1*-2	CACCGACTCGTCTTTTAACTACCCT	AAACAGGGTAGTTAAAAGACGAGTC
g*PSMC1*-1	CACCGCCCGATGTCAGTAGGAACCT	AAACAGGTTCCTACTGACATCGGGC
g*PSMC1*-2	CACCGTGACACCTCACACTCAGTGC	AAACGCACTGAGTGTGAGGTGTCAC
g*FAF2*-1	CACCGCAATCCCCCTACGCTACAGG	AAACCCTGTAGCGTAGGGGGATTGC
g*FAF2*-2	CACCGGGATCAGTGTCGCCATACCT	AAACAGGTATGGCGACACTGATCCC
g*MFF*-1	CACCGCTCAGCGTAAGTACACGAGG	AAACCCTCGTGTACTTACGCTGAGC
g*MFF*-2	CACCGTAGTGTGATAATGCAAGTTC	AAACGAACTTGCATTATCACACTAC
g*FIS1*-1	CACCGAAGGCCTTAAAGTACGTCCG	AAACCGGACGTACTTTAAGGCCTTC
g*FIS1*-2	CACCGTTACGTCTTCTACCTGGCCG	AAACCGGCCAGGTAGAAGACGTAAC
g*MID51*-1	CACCGACTTTGTGCTCTCCAATGCC	AAACGGCATTGGAGAGCACAAAGTC
g*MID51*-2	CACCGTCGAAGGTGGAGGAGTCTGT	AAACACAGACTCCTCCACCTTCGAC
g*MID49*-1	CACCGCTGTTTGGCCAAAGCCACCT	AAACAGGTGGCTTTGGCCAAACAGC
g*MID49*-2	CACCGCAGAGTTCTCCCAGAAACGG	AAACCCGTTTCTGGGAGAACTCTGC
g*DRP1*-1	CACCGAAATAGCTACGGTGAACCCG	AAACCGGGTTCACCGTAGCTATTTC
g*DRP1*-2	CACCGGAAGATAAACGGAAAACAAC	AAACGTTGTTTTCCGTTTATCTTCC
g*US30*-1	CACCGAACGGTGCGACGTTCTCCAA	AAACTTGGAGAACGTCGCACCGTTC
g*US30*-2	CACCGGTTGACGATGCCGCAATTCG	AAACCGAATTGCGGCATCGTCAACC
